# Maresin1 improves cardiac dysfunction via LGR6 in heatstroke

**DOI:** 10.1038/s12276-026-01853-6

**Published:** 2026-10-02

**Authors:** Ya-ting Jiang, Chen-xi Shen, Suwas Bhandari, Li-ran Su, Fang-jing Ni, Shuang-shuang Li, Bo Feng, Wen-yan Zhang, Lin-feng Liu, Jun Luo, Xiao-dong Pan, Yu-jie Zhou, Hui Li, Yu-qiang Gong, Sheng-wei Jin, Yu Hao

**Affiliations:** 1 Department of Anaesthesia and Critical Care, The Second Affiliated Hospital and Yuying Children’s Hospital of Wenzhou Medical University, Key Laboratory of Pediatric Anesthesiology, Ministry of Education, Wenzhou Medical University, Key Laboratory of Anesthesiology of Zhejiang Province, Wenzhou Medical University, Wenzhou, Zhejiang China; 2 Department of Critical Care Medicine, Wenzhou People’s Hospital, Wenzhou, Zhejiang China; 3 Department of Pharmacy, Taizhou Women and Children’s Hospital of Wenzhou Medical University, Taizhou, Zhejiang China; 4 Department of Emergency Medicine, The Second Affiliated Hospital and Yuying Children’s Hospital of Wenzhou Medical University, Wenzhou, Zhejiang China; 5 Wenzhou Key Laboratory of Precision General Practice and Health Management, Wenzhou, Zhejiang China

**Keywords:** Acute inflammation, Experimental models of disease

## Abstract

Heatstroke is a severe and life-threatening condition that can lead to cardiac dysfunction and poses a high risk of mortality. Maresin1, an endogenous lipid mediator, reportedly promotes inflammation resolution in various inflammatory diseases. However, its cardioprotective role during heatstroke has not been thoroughly investigated. In this study, we used ultra-performance liquid chromatography–tandem mass spectrometry to measure the levels of Maresin1 in the plasma and cardiac tissues of mice exposed to heatstroke. Our results indicated a notable decrease in Maresin1 levels under heatstroke conditions. In addition, the levels of leucine-rich repeat-containing G-protein-coupled receptor 6 (LGR6), a receptor for Maresin1, were increased in mice exposed to heatstroke. Exogenously administered Maresin1 significantly improved survival rates and alleviated the symptoms of heatstroke. Conversely, in *Lgr6*-knockout mice, the symptoms worsened, and the mortality rate increased. Transcriptomic analysis of the cardiac tissue and in vitro experiments have confirmed that Maresin1 exerts cardioprotective effects by inhibiting oxidative stress and promoting ATP synthesis through the upregulation of peroxisome proliferator-activated receptor-γ coactivator 1α (PGC1α) mediated via LGR6. Nuclear respiratory factor 1 (NRF1) and nuclear respiratory factor 2 (NRF2) were also increased downstream, likely as a secondary response associated with improved mitochondrial biogenesis rather than direct transcriptional regulation by LGR6. Our findings indicate that the Maresin1–LGR6 axis serves a cardioprotective role in heatstroke and is a potential therapeutic target for heatstroke-induced cardiac dysfunction.

## Maresin1 enhances cardiac protection during heatstroke events

Heatstroke, a severe condition marked by extreme body temperature and organ failure, poses significant health risks, particularly to the heart. This study explores the role of Maresin1, a lipid mediator known for its anti-inflammatory properties, in protecting the heart during heatstroke. Researchers used a mouse model to simulate heatstroke conditions and administered Maresin1, observing its effects on cardiac function. They found that Maresin1 significantly reduced cardiac injury markers and improved cardiac function by alleviating oxidative stress and preserving mitochondrial integrity. The study highlights the interaction between Maresin1 and the LGR6 receptor, showing that the protective effects of Maresin1 are mediated through this receptor. These findings suggest that the Maresin1–LGR6 axis is a promising target for developing treatments for heatstroke-induced cardiac injury. Future research could focus on optimizing Maresin1 dosing and timing to enhance its therapeutic potential.

This summary was initially drafted using artificial intelligence, then revised and fact-checked by the author.

## Introduction

Heatstroke (HS), a life-threatening condition, is characterized by severe hyperthermia exceeding 40.5 °C accompanied by multiorgan dysfunction, with a high mortality rate^1–3^. HS-induced cardiovascular dysfunction is a major contributor to mortality in HS cases^4–7^. Currently, clinical practice mainly uses electrocardiograms, cardiac ultrasound, and biological markers of myocardial injury to evaluate the severity of myocardial damage caused by HS^4,5,8^. Despite the severity and clinical implications of HS-related cardiac injury, effective treatment strategies beyond cooling, circulatory support, and anti-inflammatory interventions remain elusive^9^.

In recent years, the role of lipid mediators in cardiovascular protection has gained remarkable attention. Maresin1, a naturally occurring lipid mediator derived from omega-3 polyunsaturated fatty acids, has been recognized for its anti-inflammatory and cardioprotective properties. It has demonstrated therapeutic potential in various disease models, including pulmonary hypertension, cardiovascular conditions, neurodegenerative diseases, and metabolic disorders^10–16^. Maresin1 reportedly improves ventricular remodeling and arrhythmias in mice with myocardial infarction by activating antioxidant pathways and inhibiting inflammatory signaling pathways, for example, reducing the release of inflammatory cytokines, decreasing cardiomyocyte death, and mitigating interstitial fibrosis^14,17^. However, it remains unclear whether Maresin1 acts through specific receptors in cardiomyocytes, vascular endothelial cells, or immune cells. Previous studies have primarily focused on its anti-inflammatory effects mediated through receptors such as ALX/FPR2. However, evidence demonstrating whether these receptors directly mediate their cardioprotective effects remains lacking. Furthermore, in various cardiovascular diseases (e.g., myocardial infarction, heart failure, and diabetic cardiomyopathy), whether endogenous Maresin1 levels are sufficient to exert physiological regulatory effects at different stages of the disease remains unclear; similarly, the optimal timing and dosing strategies for exogenous Maresin1 supplementation have yet to be been determined. The precise mechanisms by which Maresin1 exerts its cardioprotective effects, particularly in the context of HS, remain underexplored.

LGR6, a G-protein-coupled receptor containing leucine-rich repeats, plays a vital role in tissue repair and regulation of cellular metabolism^13,18^. Importantly, the biological functions of many G-protein-coupled receptors, including LGR6, are highly context-dependent^19^. Although their constitutive expression may contribute modestly to basal cellular homeostasis, pathological stress conditions can sensitize receptor activity and amplify downstream signaling pathways, thereby serving as critical adaptive mechanisms to protect cells from injury. Recent studies have demonstrated that cardiomyocyte LGR6 overexpression exerts protective effects against ischemia–reperfusion injury^20^. Cardiomyocyte LGR6 regulates mitochondrial biogenesis and alleviates ferroptosis in diabetic cardiomyopathy, uncovering a novel therapeutic mechanism for managing cardiomyopathies^21^.

Emerging evidence suggests that Maresin1 exerts cardioprotective effects through its interaction with LGR6^22,23^, highlighting the potential therapeutic significance of the Maresin1–LGR6 axis. In our research, we show that the Maresin1–LGR6 axis plays a cardioprotective role in HS. The absence of Maresin1 and deletion of LGR6 exacerbate myocardial injury symptoms in HS, whereas exogenous Maresin1 administration demonstrates a protective effect against HS through LGR6. We first performed single-nucleus RNA sequencing (RNA-seq) on cardiac tissues from mice exposed to HS, revealing that Maresin1 protects HS-induced myocardial injury by alleviating oxidative stress and preserving mitochondrial function. These findings indicate that the Maresin1–LGR6 axis is a potential therapeutic target for HS-induced cardiac injury.

The existing knowledge gap regarding the role of Maresin1 in cardiovascular diseases, particularly in the context of HS, has constrained its potential clinical applications. Our findings suggest that the Maresin1–LGR6 axis serves as a novel therapeutic target for preventing or treating HS-induced cardiac injury, thereby presenting a promising pathway for future cardiovascular interventions.

## Materials and methods

### Heatstroke model

The HS model was developed following established protocols^24^. In the HS group, mice were exposed to HS by being placed in a climate chamber (YAN-T1000L, YUYAN) at 39 °C and 60 ± 5% relative humidity for 2 hours. Core body temperature was recorded every 15 minutes using a rectal thermocouple (BW-TH1101, Biowill, Shanghai, China).

After exposure to HS, the mice were returned to their home cages, maintained at 25 °C, and had free access to food and water. Conversely, in the sham group, mice underwent identical procedures without being exposed to HS.

### Maresin1 administration

Maresin1 (Mar1, 10878, Cayman) was dissolved in saline and administered via tail vein injection after the HS model was established. The administration dose (2, 4, 8, and 10 mg/kg) was determined based on prior research into its efficacy in treating inflammatory conditions.

### Isolation of primary mouse cardiomyocytes

Following the previously described procedure^25^, postnatal day 3–5 mice were deeply anesthetized using isoflurane inhalation to ensure complete sedation throughout the procedure. The hearts were promptly excised, rinsed twice in chilled Hank’s Balanced Salt Solution (HBSS), and cut into sections. Next, they were processed in a collagenase and elastase solution at 37 °C for 35 minutes. The cell suspension output was transferred to a cardiomyocyte-specific culture medium supplemented with 10% fetal bovine serum to stop the digestion. The supernatant from the mixture, centrifuged at 500 *g* for 5 minutes, was discarded. The cell pellet was resuspended in HBSS, washed twice, and carefully pipetted with a 1000 µl pipette to create a single-cell suspension free of visible clumps. Cell concentration was measured, and cells were seeded at a density of 1 ×  10^6^ cells/ml. The medium was gently replaced with a fresh one to avoid disturbing the adherent cells 24 hours post-incubation and subsequently changed every 48 hours.

### Cell culture and experimental treatments

Primary cardiomyocytes were cultured using the iCell Primary Cardiomyocyte Culture System (iCell Bioscience). The cells were maintained at 37 °C with 5% CO₂ for 24 hours, reaching approximately 90% confluence. The cells were exposed to 39 °C for 2 hours and incubated at 37 °C with 5% CO₂ for 12 hours to establish an HS cell model. After HS induction, cells were treated with Maresin1 by replacing the culture medium with fresh medium containing various concentrations of Maresin1. The treated cells were then returned to a standard incubator for 12 hours.

### Statistical analysis

Data are presented as mean ± s.e.m., with significance set at *P* < 0.05, and analyzed using GraphPad Prism 10.0. All data were subjected to a normality test before statistical analysis, using the Shapiro–Wilk test (or Kolmogorov–Smirnov test) to assess whether the data followed a normal distribution. Body temperature curves were evaluated using repeated measures analysis of variance (ANOVA), followed by Bonferroni post hoc multiple comparisons. Survival curves were analyzed using the log-rank test. Results with *P *< 0.05 were considered significant. Two-group comparisons used a two-tailed unpaired Student’s *t*-test, while three-group analyses used a one-way ANOVA with Tukey’s post hoc test. Statistical significance is indicated as follows: ns, no significant difference; *P < 0.05; **P < 0.01; ***P < 0.001.

The Supplementary Methods include detailed animal preparation, liquid chromatography–tandem mass spectrometry construction protocols, RNA-seq analysis, transthoracic echocardiography, enzyme-linked immunosorbent assay (ELISA), biochemical assays, heart histology, and immunofluorescence. Additional techniques, such as transmission electron microscopy (TEM), real-time quantitative PCR (RT-qPCR), mouse cardiac tissue flow cytometry, and measurements of ATP and malondialdehyde (MDA) content, are also described. Methods for assessing mitochondrial membrane potential, reactive oxygen species (ROS) levels, mitochondrial stress, small interfering RNA (siRNA) transfection, and western blot analysis are also provided^25^.

## Results

### Mice exposed to heatstroke showed myocardial damage and diastolic dysfunction

**Fig. 1:**
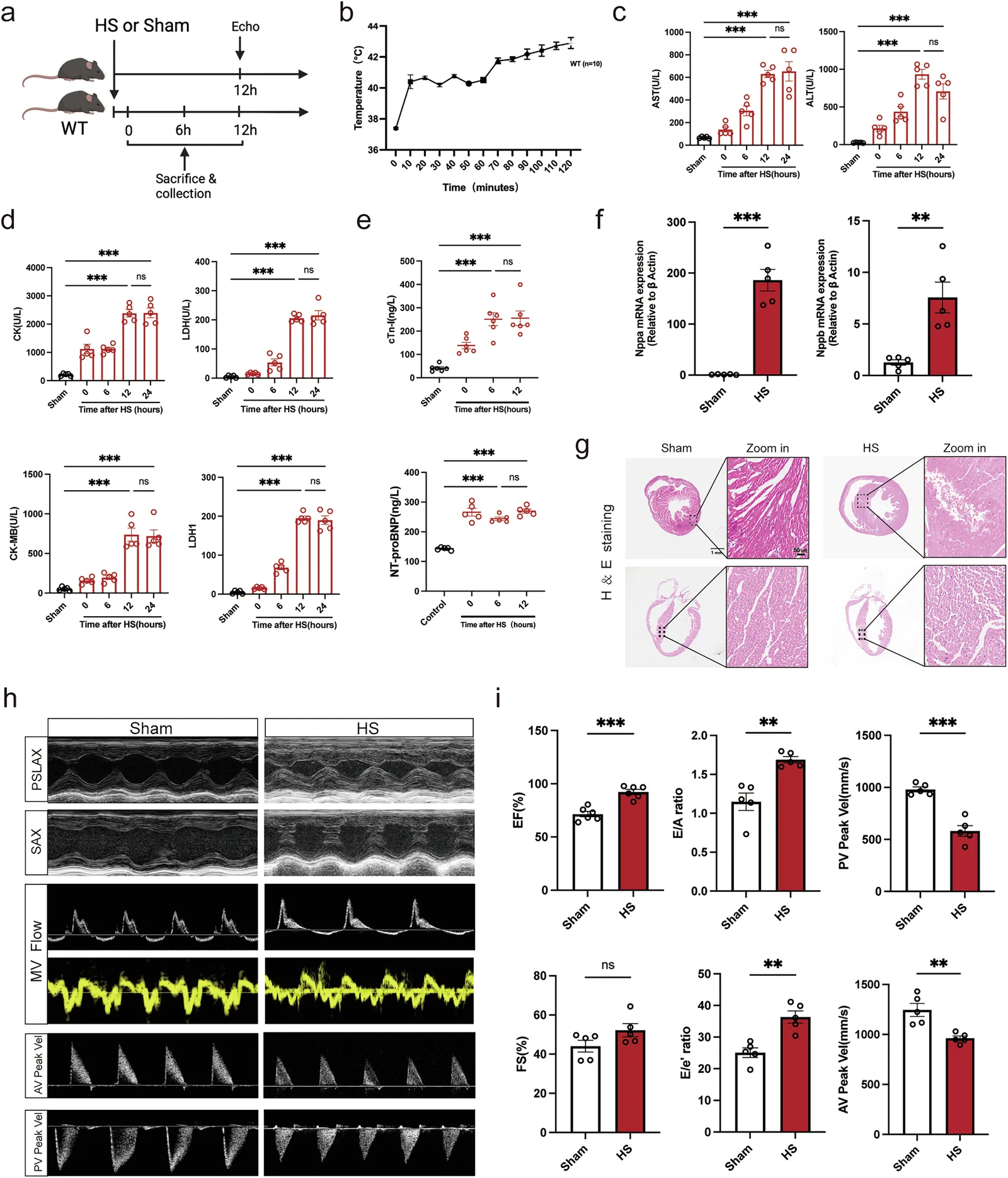
HS-induced myocardial damage and diastolic dysfunction. **a** Timeline illustrating the construction of the mouse model. **b** Core body temperatures of mice exposed to HS (*n* = 10). **c** Plasma levels of AST and ALT (*n* = 10). **d** CK, CK-MB, LDH, and LDH1 plasma concentrations at 0, 6, 12 and 24 hours after HS (*n* = 5). **e** cTn-I and NT-proBNP plasma levels measured at 0, 6, and 12 hours after HS (*n* = 6). **f** RT-qPCR analysis of Nppa and Nppb gene expression in cardiac tissues after sham or HS conditions. **g** Hematoxylin and eosin (H&E) staining of cardiac tissue sections (scale bar = 50 μm). **h** Representative echocardiographic images from mice exposed to HS. **i** Quantifying echocardiographic parameters, including ejection fraction (EF), fractional shortening (FS), E/e’ ratio, E/A ratio, aortic valve (AV) peak velocity, and pulmonary vein (PV) peak velocity in mice exposed to HS (*n* = 6). Statistical analyses were performed using repeated measures ANOVA, followed by Bonferroni post hoc multiple comparisons (**b**) using one-way ANOVA with Tukey’s multiple comparisons test (**c–e**) and using a two-tailed unpaired Student’s *t*-test (**f,i**). Data are presented as mean ± s.e.m.. Statistical significance is indicated as follows: ns, no significant difference; *P < 0.05, **P < 0.01, ***P < 0.001.

Wild-type (WT) mice were exposed to 39°C in a chamber with 60 ± 5% relative humidity for 2 hours to induce HS (Fig. 1a). Core body temperature and plasma markers of organ injury were monitored over time. The rectal temperature results demonstrated that HS raised the core body temperature to 43 °C (Fig. 1b). Infrared thermography results showed that the temperature in the cardiac region of the mice reached approximately 39 °C (Supplementary Fig. 7). Furthermoree, ELISA results indicated significantly increased plasma levels of the liver injury markers AST and ALT, particularly 12 hours after HS (Fig. 1c,d). Plasma biomarkers indicative of cardiac injury, including CK, LDH, CK-MB, LDH1, cTn-I and NT-proBNP, were significantly elevated at 12 hours after HS, indicating myocardial injury (Fig. 1e). Similarly, the expression of cardiac stress markers, Nppa and Nppb, showed comparable increases in the HS group (Fig. 1f). To further investigate the mechanisms underlying myocardial dysfunction during HS, histological analysis was performed (Fig. 1g). Echocardiographic analysis demonstrated a progressive decline in cardiac function during HS. Diastolic dysfunction was identified by elevated E/A (ratio of peak early to late transmitral flow velocities) and E/e’ (ratio of early transmitral flow to early diastolic mitral annular velocity) ratios, as well as reduced aortic and pulmonary arterial flow velocities. Notably, significant impairments became evident 12 hours after the onset of HS (Fig. 1h,i). Collectively, these findings implied that HS induces severe myocardial injury and diastolic dysfunction.

### Dysregulation of the Maresin1–LGR6 axis in heatstroke

**Fig. 2:**
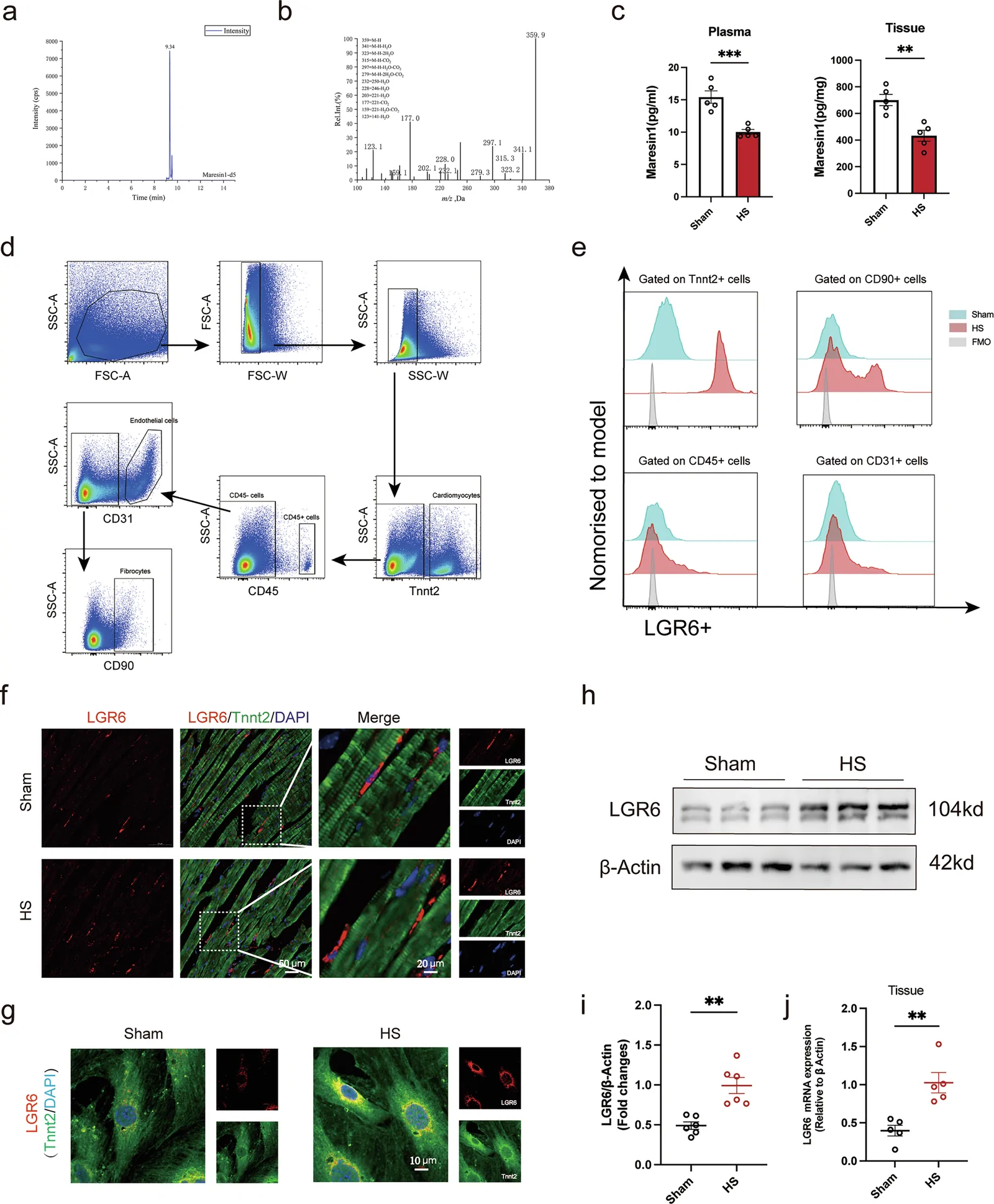
Dysregulation of the Maresin1–LGR6 axis in HS. **a** Multiple reaction monitoring chromatograms showing the retention time for Maresin1 (m/z 375/141). **b** MS/MS spectrum of Maresin1. **c** Quantifying Maresin1 levels in the plasma and cardiac tissues from sham and HS mice at 6 and 12 hours after HS (*n* = 5). **d**,**e** Flow cytometric analysis and quantification of various cellular populations in cardiac tissues, with specific markers identifying endothelial cells (CD31), immune cells (CD45), cardiomyocytes (Tnnt2), and fibroblasts (CD90). **f** Representative immunofluorescence staining of cardiac tissues showing LGR6 (red), Tnnt2 (green), and DAPI (blue) (scale bars = 50 μm and 20 μm; *n* = 4). **g** Representative immunofluorescence images of primary mouse cardiomyocytes stained for LGR6 (red), Tnnt2 (green), and DAPI (blue) (scale bar = 10 μm; *n* = 4). **h**,**i** Western blot analysis and quantification of LGR6 expression in cardiac tissues from sham and HS mice (*n* = 6). **j** RT-qPCR analysis of LGR6 gene expression in cardiac tissues from sham and HS mice (*n* = 5). Statistical analyses were performed using a two-tailed unpaired Student’s *t*-test (**c**,**i**,**j**). Data are presented as mean ± s.e.m. Statistical significance is indicated as follows: ns, no significant difference; *P < 0.05, **P < 0.01, ***P < 0.001.

We analyzed samples from an HS mouse model 12 hours after HS to identify and quantify plasma and cardiac tissue lipid mediators (Fig. 2a,b). Among the lipid mediators, Maresin1 levels were significantly reduced 12 hours after HS, hinting a potential role for Maresin1 depletion in the progression of HS-induced cardiac dysfunction (Fig. 2c). Furthermore, to investigate the cellular targets of Maresin1 via its receptor LGR6, we performed flow cytometry on cardiac tissues from sham and HS groups (Fig. 2d). The analysis demonstrated that LGR6 was primarily localized to cardiomyocytes and was markedly upregulated in HS-treated cardiomyocytes compared with sham (Fig. 2e). In vitro, we modeled HS in primary mouse cardiomyocytes by exposing them to 39 °C for different hours to simulate hyperthermic conditions. The heat exposure caused severe cardiomyocyte damage, as evidenced by a marked increase in Nppa expression observed 2 hours after HS (Supplementary Fig. 4a). Furthermore, immunofluorescence (Fig. 2f,g), western blot (Fig. 2h), and PCR (Fig. 2i,j) analyses confirmed that LGR6 expression in both cardiomyocytes and cardiac tissues was significantly elevated under HS. The results suggest a significant role of the Maresin1–LGR6 axis in mediating cardiac protection during HS, warranting further investigation into its therapeutic implications.

### Maresin1 treatment improves cardiac function and survival in heatstroke

**Fig. 3:**
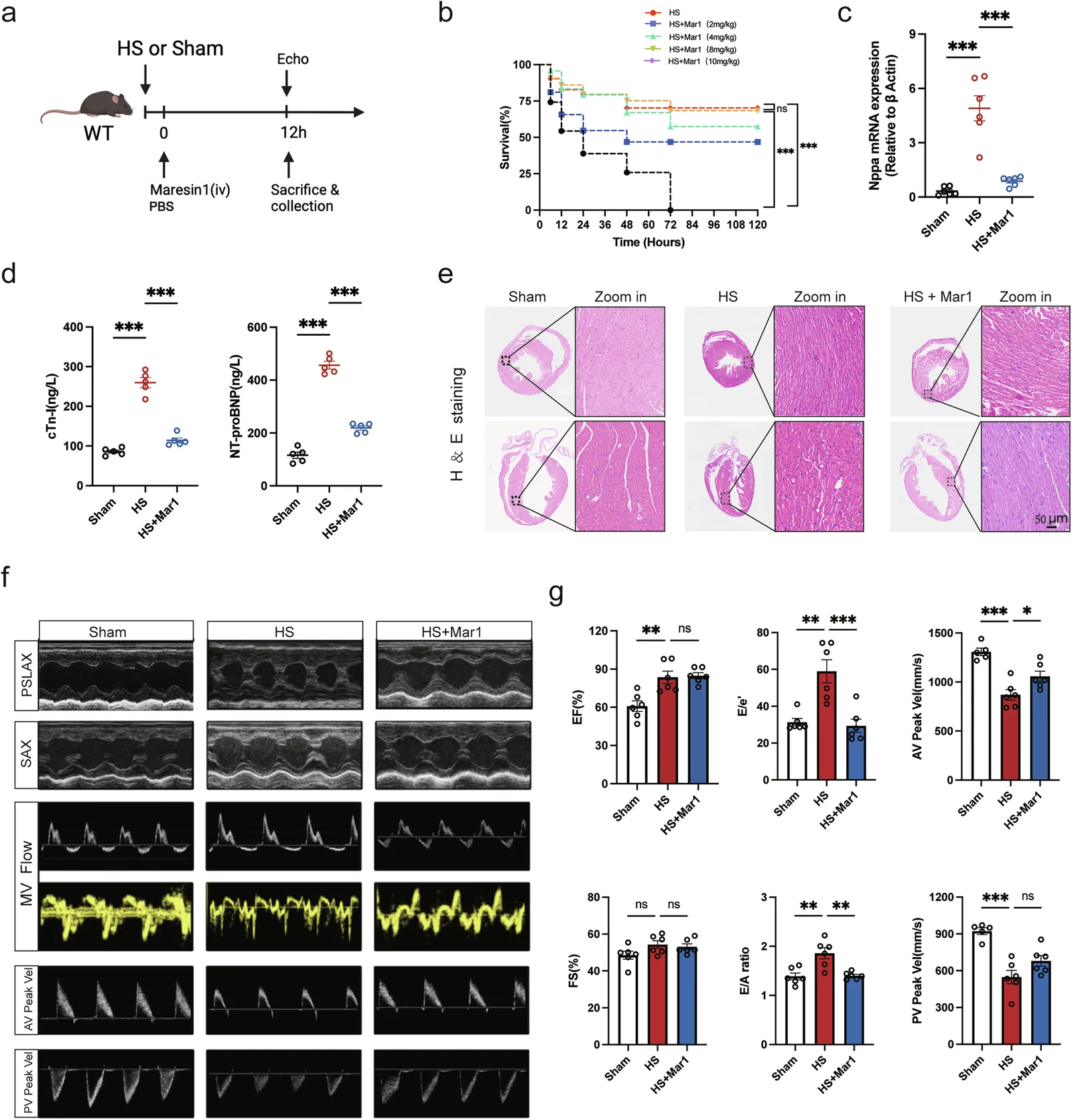
Maresin1 treatment improves cardiac dysfunction and lethality in HS. **a** Schematic representation illustrating the evaluation of Maresin1 effects on HS mice. **b** Kaplan–Meier survival curves illustrating the effect of Maresin1 treatment on survival after HS (*n* = 16). **c** RT-qPCR analysis of Nppa mRNA expression in cardiac tissues (*n* = 6). (d) Plasma concentrations of cTn-I and NT-proBNP (*n* = 5). **e** H&E staining of cardiac tissue sections from HS mice treated with Maresin1 (scale bar = 50 μm). **f** Representative echocardiographic images of the left ventricle (LV) in HS mice treated with Maresin1. **g** Quantification of echocardiographic parameters, including EF, FS, E/e’ ratio, E/A ratio, AV peak velocity, and PV peak velocity, in HS mice treated with Maresin1 (*n* = 6). Statistical analyses: survival data were analyzed using the log-rank (Mantel–Cox) test, with results presented as survival percentages over time (hours) on the x-axis (**b**); analyzed using one-way ANOVA, followed by Tukey’s multiple comparisons test (**c**,**d**,**g**). Data are expressed as mean ± s.e.m.. Statistical significance is indicated as follows: ns, no significant difference; *P < 0.05, **P < 0.01, ***P < 0.001.

To elucidate the impact of Maresin1 on HS, mice exposed to HS were administered varying doses of Maresin1, and their survival rates were analyzed (Fig. 3a,b). No significant difference was found between the 8 and 10 mg/kg doses. Maresin1 treatment significantly reduced myocardial injury biomarkers in HS-induced cardiac dysfunction (Fig. 3d). The myocardial injury marker Nppa was notably reduced at the 8 mg/kg dose (Fig. 3c). On H&E staining, HS resulted in apparent cytoplasmic vacuolation in cardiomyocytes, which was markedly improved with Maresin1 treatment (Fig. 3e). Echocardiographic analysis revealed that Maresin1 treatment improved left ventricular diastolic dysfunction and cardiac blood flow in HS mice, indicated by decreased E/A and E/e’ ratios and enhanced aortic and pulmonary arterial flow velocities (Fig. 3f,g). These findings suggest that Maresin1 effectively attenuates HS-induced cardiac dysfunction at an optimal therapeutic dose of 8 mg/kg.

### Maresin1 exerts cardioprotective effects via LGR6 in heatstroke mice

**Fig. 4:**
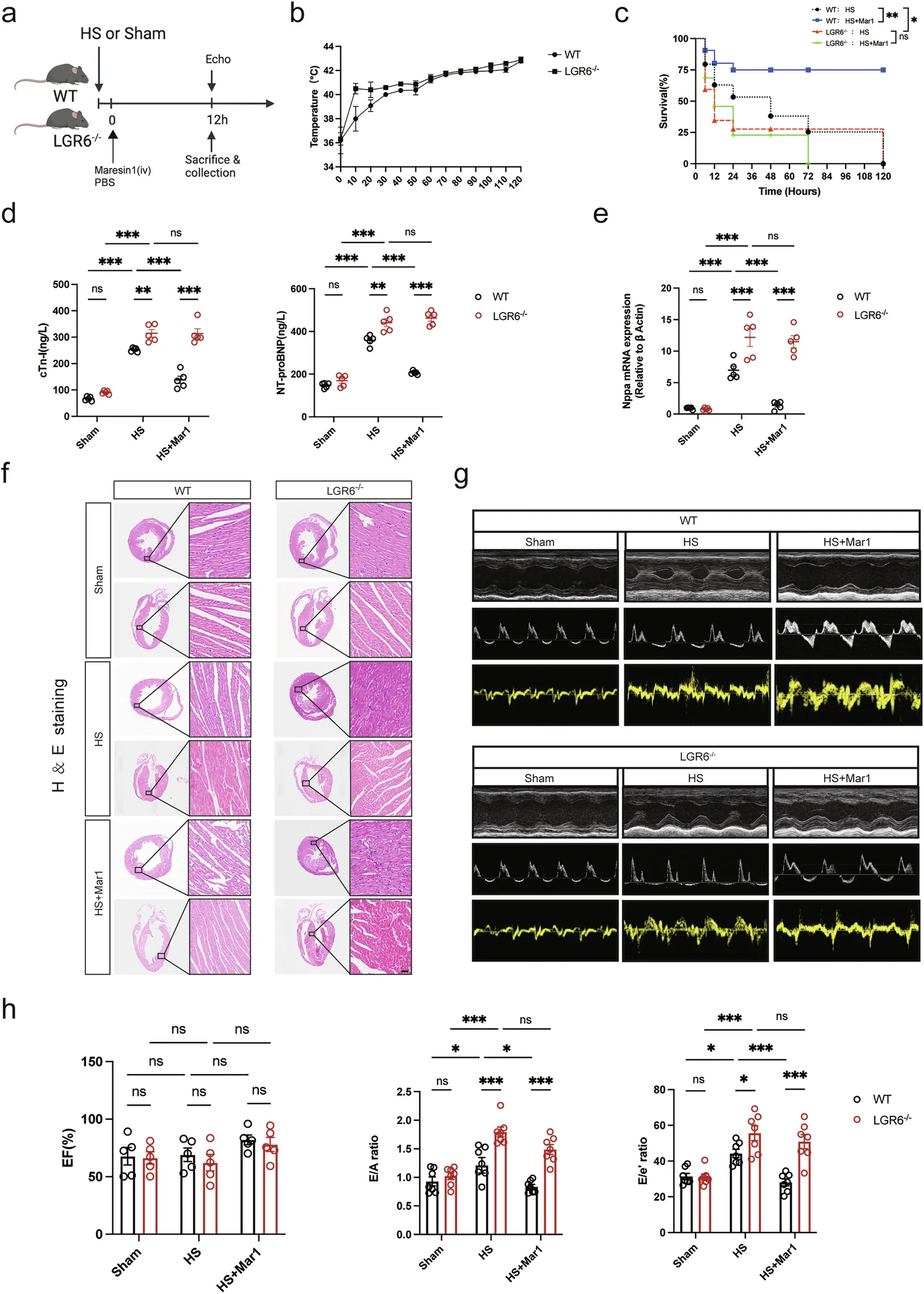
Maresin1 exerts protective effects via LGR6 in HS mice. **a** Diagram depicting the experimental strategy. **b** Core body temperatures of mice exposed to HS (*n* = 10). **c** Kaplan–Meier survival curves exhibit the Maresin1 treatment effect on survival after HS (*n* = 16). **d** Plasma concentrations of cTn-I and NT-proBNP in WT and *Lgr6*-knockout (*Lgr6*^−/−^) mice (*n* = 5). **e** RT-qPCR analysis of Nppa expression in cardiac tissues (*n* = 5). **f** H&E staining of heart sections from WT and *Lgr6*^−/−^ mice exposed to HS with or without Maresin1 treatment (scale bars = 1 mm and 50 μm). **g** Representative echocardiographic images. **h** Quantification of echocardiographic parameters, including EF, FS, E/e’ ratio, E/A ratio, AV peak velocity, and PV peak velocity, in HS mice (*n* = 5). Statistical analyses: analyzed using repeated measures ANOVA, followed by Bonferroni post hoc multiple comparisons (**b**); survival data were analyzed using the log-rank (Mantel–Cox) test, and results were presented as survival percentages over time (hours) on the x-axis (**c**); analyzed using two-way ANOVA with Tukey’s multiple comparisons test (**d**,**e**,**h**). Data are expressed as mean ± s.e.m.. Statistical significance is indicated as follows: ns, no significant difference; *P < 0.05, **P < 0.01, ***P < 0.001.

Maresin1 exerts its pro-resolving effects through the LGR6 receptor. Administration of Maresin1 alone resulted in an increase in LGR6 expression in cardiac tissues (Supplementary Fig. 9a,b). To investigate whether Maresin1, via LGR6, improves cardiac dysfunction in HS mice in vivo, we generated global *Lgr6*-knockout (*Lgr6*^−/−^) mice, which were confirmed to be homozygous (Supplementary Fig. 1). Under sham conditions, *Lgr6*^−/−^ mice exhibited normal cardiac function and histological morphology comparable with WT mice. However, the context-dependent protective role of LGR6 became evident under HS. *Lgr6*^−/−^ mice displayed exacerbated cytoplasmic vacuolation (Fig. 4f,g). Subsequently, *Lgr6*^−/−^ mice were exposed to HS and treated with Maresin1 (Fig. 4a). The body temperature curves showed no notable differences, and survival analysis indicated that therapeutic effects of Maresin1 were absent in *Lgr6*^−/−^ mice (Fig. 4b,c). Plasma concentrations of AST, ALT, and cardiac biomarkers (CK-MB, LDH1, cTn-I and NT-proBNP) were significantly elevated in the HS group compared with the sham group. In the HS+Maresin1 (HS+Mar1) group compared with WT mice, these biomarker levels showed no significant differences (Fig. 4d,e and Supplementary Fig. 2). Histological and cardiac functional analysis of *Lgr6*^−/−^ mice compared with WT mice revealed no significant differences in H&E staining or echocardiographic parameters under sham conditions. However, after HS, *Lgr6*^−/−^ exacerbated cytoplasmic vacuolation in cardiomyocytes (Fig. 4f). Echocardiographic analysis further demonstrated that *Lgr6*^−/−^ aggravated cardiac dysfunction after HS (Fig. 4g,h). Notably, administration of Maresin1 did not significantly improve these outcomes in *Lgr6*^−/−^ mice. These findings suggest that LGR6 deficiency aggravates myocardial injury in HS mice, regardless of Maresin1 treatment.

### Cardiomyocyte-specific LGR6 knockdown accelerates cardiac dysfunction in heatstroke

**Fig. 5:**
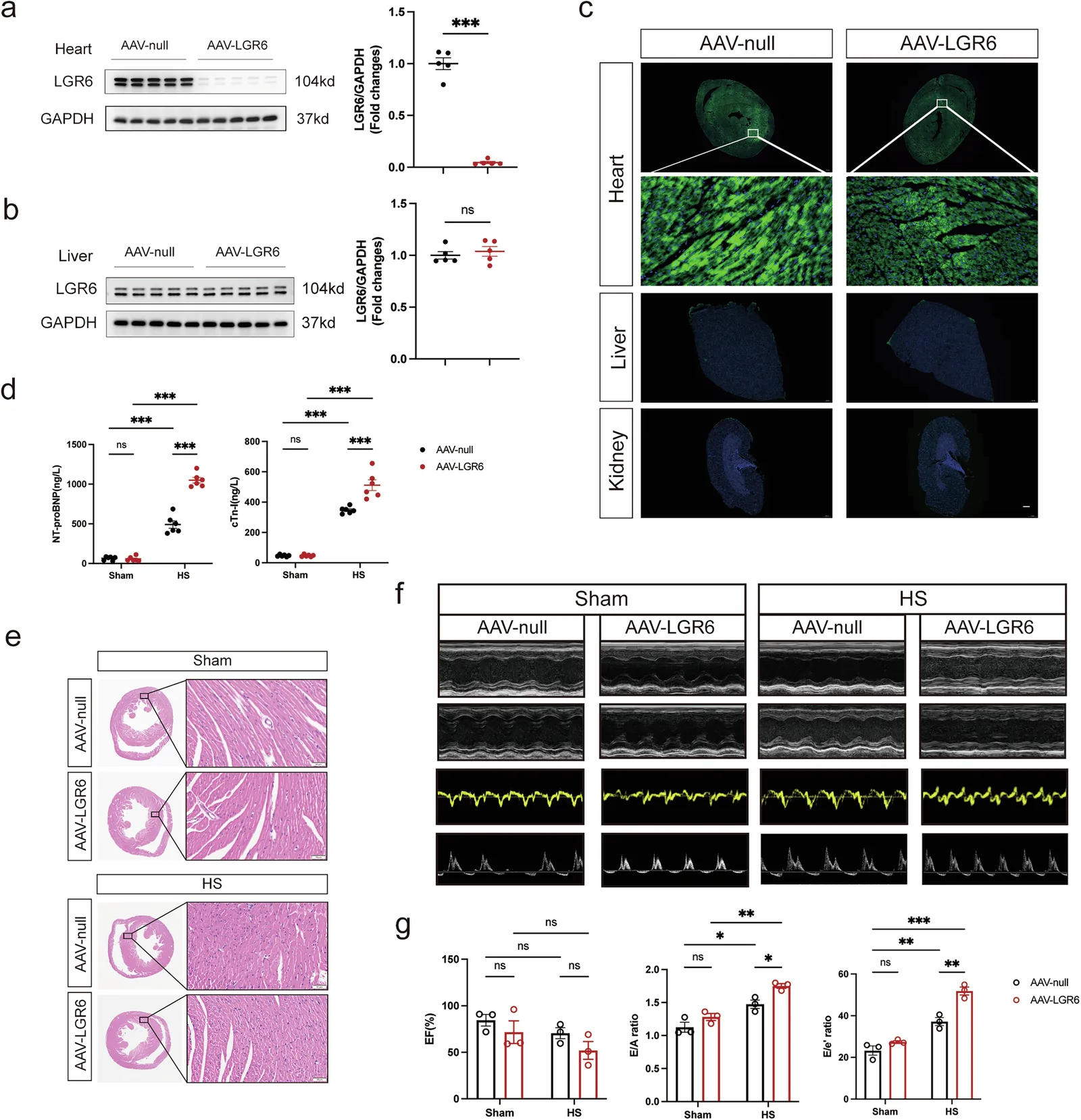
Cardiomyocyte-specific LGR6 knockdown accelerates cardiac dysfunction in HS. **a**,**b** Immunoblot analysis of LGR6 expression in the heart and liver tissues of control AAV (AAV-null) and AAV-LGR6 mice (*n* = 5). **c** Immunofluorescence staining of heart, liver, and kidney tissues showing LGR6 expression (green) in AAV-null and AAV-LGR6 mice. Nuclei are stained with DAPI (blue). **d** Plasma ELISA results showing cTn-I and NT-proBNP levels in AAV-null and AAV-LGR6 mice under sham and HS conditions (*n *= 6). **e** H&E staining of cardiac tissue sections from sham and HS groups, showing myocardial injury in HS mice. **f**,**g** Echocardiography images and quantification of cardiac function in sham and HS conditions for AAV-null and AAV-LGR6 mice (*n* = 3). Statistical analyses were performed using a two-tailed unpaired Student’s *t*-test (**a**,**b**) and using two-way ANOVA followed by Tukey’s multiple comparisons test (**d**,**g**). Data are presented as mean ± s.e.m.. Statistical significance is indicated as follows: ns, no significant difference; *P < 0.05; **P < 0.01; ***P < 0.001.

In this study, we investigated the impact of cardiomyocyte-specific LGR6 knockdown on cardiac function during HS. Using a cardiomyocyte-specific *Lgr6*-knockout mouse model (Fig. 5a–c), we observed that the absence of LGR6 in cardiomyocytes significantly worsened cardiac dysfunction after HS. Plasma biomarker analysis indicated higher levels of cTn-I and NT-proBNP, revealing the exacerbation of myocardial damage (Fig. 5d and Supplementary Fig. 3). Histological examination of cardiac tissue further showed increased myocardial injury, with greater levels of fibrosis and inflammation in the *Lgr6*-knockout group (Fig. 5e). Furthermore, echocardiographic analysis revealed a marked reduction in EF and FS in AAV-LGR6 mice compared with AAV-null mice (Fig. 5f,g). These results suggest that cardiomyocyte-specific LGR6 expression plays a crucial role in mitigating HS-induced cardiac dysfunction, and its absence accelerates the progression of cardiac injury.

### Maresin1 mitigates HS-induced cardiac oxidative stress in vivo

**Fig. 6:**
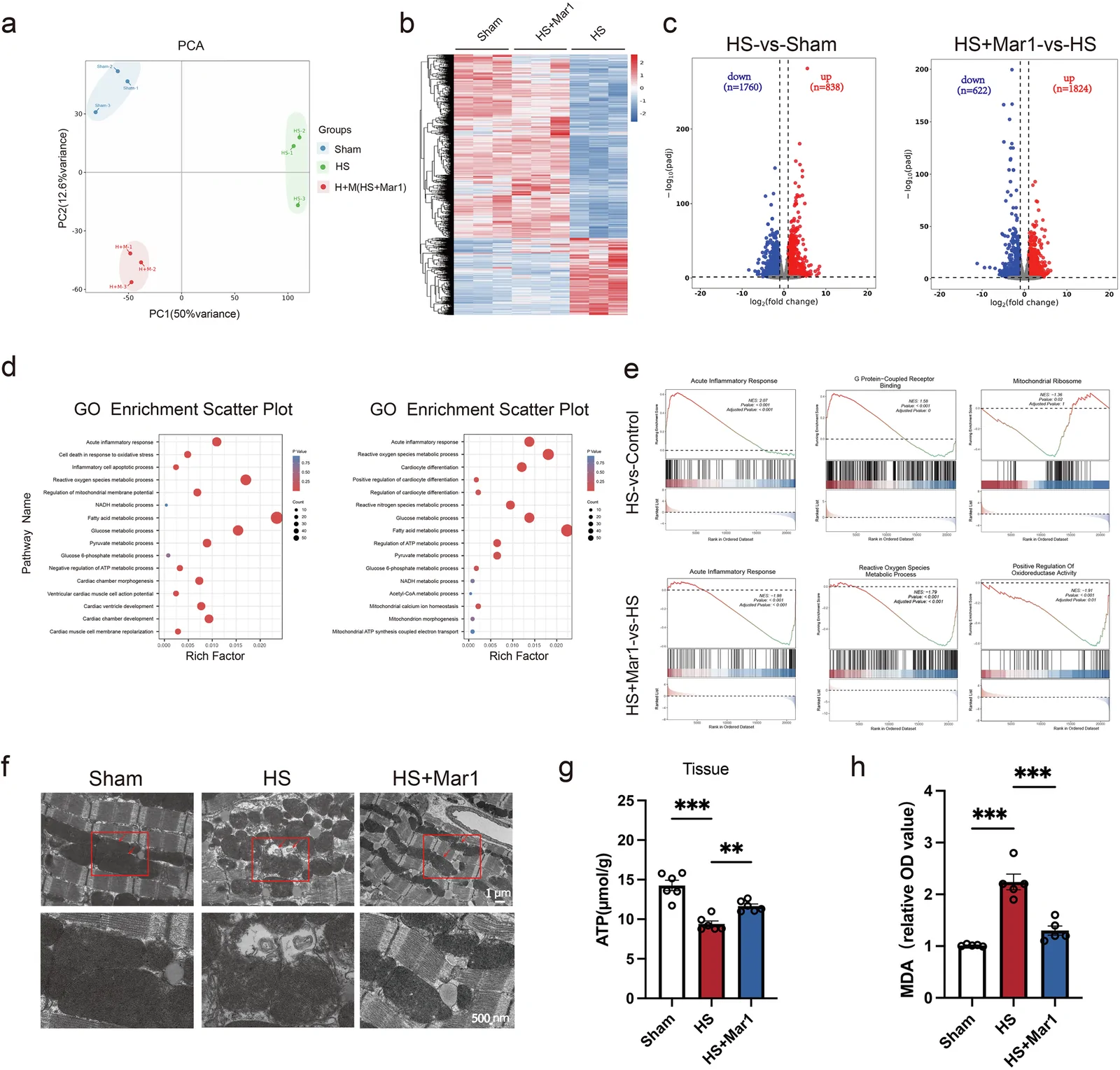
Maresin1 mitigates HS-induced cardiac oxidative stress in vivo. **a** Principal component analysis (PCA) plot illustrating the separation of clusters among the three groups (*n* = 3). **b** Genes in sham are differentially expressed by hierarchical clustering, HS, and HS+Mar1 cardiac tissues, as determined by RNA-seq (adjusted *P* < 0.05). **c** Volcano plot depicting differentially expressed genes in HS vs. sham and HS+Mar1 vs. HS cardiac tissues (log_2_fold change > 0.55, adjusted *P* < 0.05). **d** Gene Ontology enrichment analysis of RNA-seq data comparing HS vs. sham and HS+Mar1 vs. HS cardiac tissues. **e** Gene Set Enrichment Analysis plots illustrating inflammation and mitochondrial oxidative stress-related gene set enrichment. **f** TEM images of ventricular tissues from sham, HS, and HS+Mar1 mice (scale bars: low magnification = 1 μm, high magnification = 500 nm). g, ATP concentrations in cardiac tissues from sham, HS, and HS+Mar1 mice (*n* = 5). **h** MDA levels in cardiac tissues from each group (*n* = 5). Statistical analyses were performed using one-way ANOVA, followed by Tukey’s multiple comparisons test (**g**,**h**). Data are presented as mean ± s.e.m.. Statistical significance is indicated as follows: ns, no significant difference; *P < 0.05, **P < 0.01, ***P < 0.001.

To investigate the mechanisms underlying the cardioprotective effects of Maresin1 in HS-induced cardiac dysfunction, we conducted genome-wide RNA-seq on the left ventricular tissues from sham, HS, and HS+Mar1 mice. Principal component analysis confirmed high consistency among biological replicates for each group (*n* = 3, Fig. 6a). Hierarchical clustering analysis revealed that Maresin1 treatment significantly altered the cardiac transcriptome in HS mice (Fig. 6b). Between the sham and HS groups, 1,760 genes were downregulated and 838 were upregulated, with differential expression determined by an adjusted *P* < 0.05. A total of 622 downregulated and 1,824 upregulated differentially expressed genes with an adjusted *P* < 0.05 were identified between the HS and HS+Mar1 groups (Fig. 6c). Gene Ontology biological process analysis revealed notable pathway differences, including 'reactive oxygen species metabolic process' and 'regulation of ATP metabolic process', between the hearts of HS+Mar1 and HS mice (Fig. 6d). Gene Set Enrichment Analysis demonstrated that Maresin1 treatment reversed the HS-induced upregulation of the 'reactive oxygen species metabolic process' pathway, resulting in its downregulation (Fig. 6e). Considering the critical role of mitochondria in ATP production and cardiac energy balance, we used TEM to examine the structural and functional differences in hearts affected by HS and HS+Mar1. TEM analysis revealed that HS resulted in disorganized myofibrils, nuclear chromatin marginalization, and significant cytoplasmic vacuolation. Furthermore, mitochondria in cardiomyocytes exhibited swelling, cristae fragmentation or loss, and partial outer membrane rupture (Fig. 6f). HS significantly decreased ATP production; however, Maresin1 treatment maintained the mitochondrial structure and restored ATP levels (Fig. 6g). Maresin1 treatment inhibited HS-induced MDA increases in cardiac tissues (Fig. 6h). These findings demonstrate that Maresin1 treatment mitigates HS-induced mitochondrial dysfunction by preserving mitochondrial membrane potential, reducing ROS production, and normalizing mitochondrial quantity, thereby protecting cardiomyocytes from HS-induced injury.

### Maresin1 treatment alleviates cardiomyocyte mitochondrial dysfunction

**Fig. 7:**
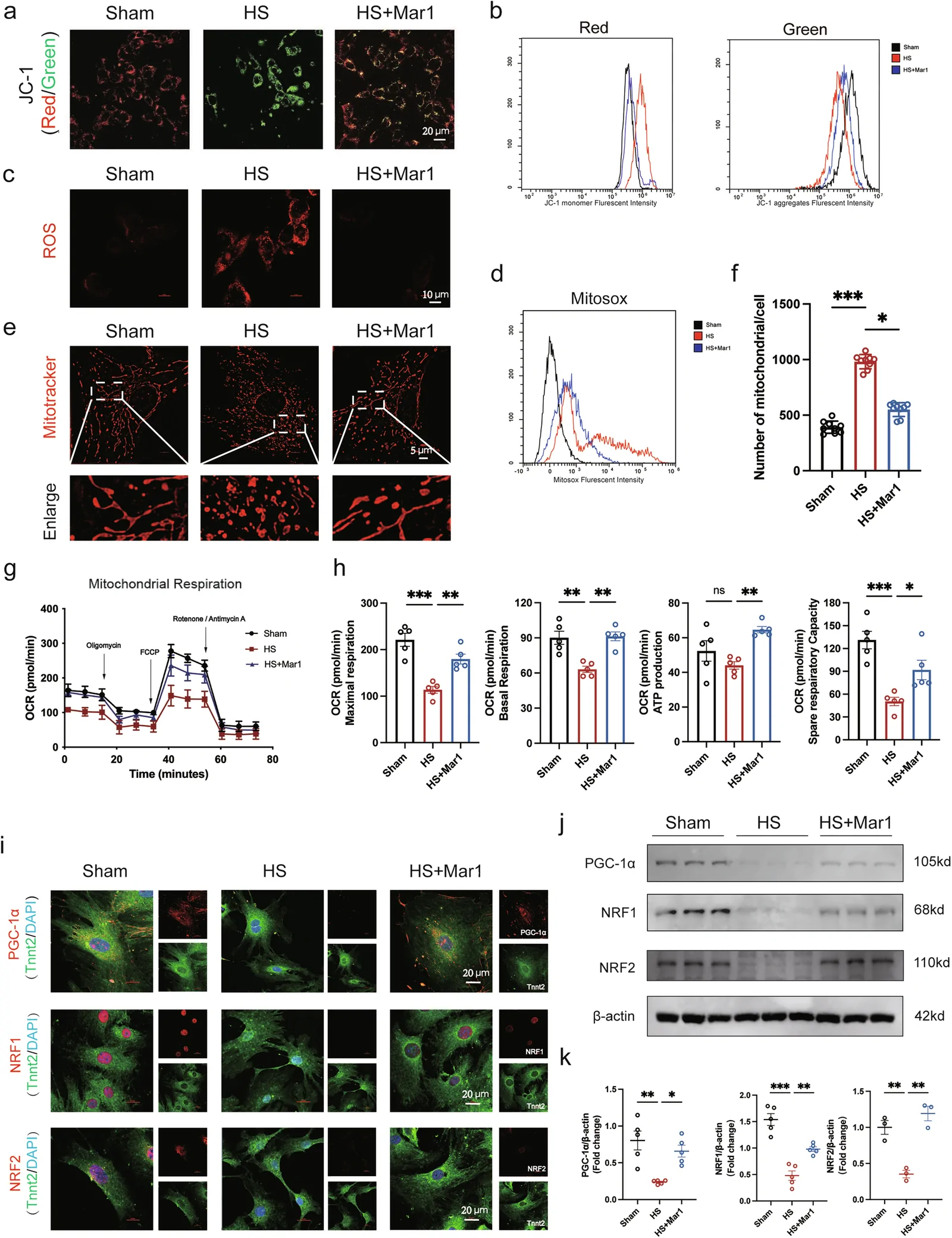
Maresin1 treatment alleviates mitochondrial dysfunction in cardiomyocytes. Primary cardiomyocytes were exposed to 39 °C for 2 hours to simulate HS, followed by treatment with Maresin1 (200 nM) for 12 hours. **a**,**b** Mitochondrial membrane potential was assessed using immunofluorescence and flow cytometry (scale bar = 20 μm; *n* = 4). **c**,**d** Mitochondrial ROS levels were evaluated in cardiomyocytes stained with MitoSOX Red and analyzed using immunofluorescence and flow cytometry (scale bar = 10 μm; *n* = 4). **e**,**f** Representative images and analysis of cardiomyocytes stained with MitoTracker Red (scale bar = 5 μm; *n* = 4). **g**,**h** Real-time monitoring of mitochondrial OCR, including a left-side OCR diagram and right-side quantification of maximal respiration, basal respiration, ATP production, and spare respiratory capacity. **i** Immunofluorescence staining for PGC1α, NRF1, and NRF2 (red), Tnnt2 (green), and DAPI (blue) in cultured cardiomyocytes from three groups (scale bar = 20 μm; *n* = 3). **j**,**k** Western blot analysis and quantification of PGC1α, NRF1, and NRF2 in cardiomyocytes (*n* = 4). Statistical analyses were performed using one-way ANOVA, followed by Tukey’s multiple comparisons test (**f**,**h**,**k**). Data are presented as mean ± s.e.m.. Statistical significance is indicated as follows: ns, no significant difference; *P < 0.05, **P < 0.01, ***P < 0.001.

Considering that Maresin1 exerts cardioprotective effects through the LGR6 receptor on cardiomyocytes, we further explored the mitochondrial function in an HS model using primary mouse cardiomyocytes. PCR analysis demonstrated that HS increased the expression of Nppa, whereas Maresin1 (200 nM) significantly alleviated cardiomyocyte damage (Supplementary Fig. 4). To further assess the impact of Maresin1 on mitochondrial oxidative stress in HS-induced cardiomyocyte injury, we performed flow cytometry and confocal microscopy. The results showed that HS led to a significant loss of mitochondrial membrane potential, elevated mitochondrial ROS levels, and increased mitochondrial quantity compared with the sham group. Maresin1 treatment notably improved mitochondrial membrane potential (Fig. 7a,b), decreased mitochondrial ROS production (Fig. 7c,d), and normalized mitochondrial abundance in cardiomyocytes (Fig. 7e,f). To further investigate mitochondrial respiratory function, we used the Seahorse XF-96 extracellular flux analyzer to assess the oxygen consumption rate (OCR) of primary mouse cardiomyocytes exposed to HS, with and without Maresin1 treatment. In cardiomyocytes exposed to HS, both maximal respiratory and ATP production capacity markedly decreased relative to the sham group (Fig. 7g,h), suggesting mitochondrial dysfunction. Mitochondrial biogenesis and its metabolic functions rely on the coordinated regulation of nuclear and mitochondrial genes. Transcription factors such as PGC1α, NRF1, and NRF2 are crucial for transcribing nuclear-encoded mitochondrial genes, aiding cellular defense against oxidative stress^26^. Immunofluorescence and western blot analyses demonstrated that Maresin1 increases the expression of PGC1α, NRF1, and NRF2 in vitro (Fig. 7i–k) and in vivo (Supplementary Fig. 9c,d). The results indicate that Maresin1 is essential for restoring mitochondrial function and alleviating HS-induced cardiomyocyte injury.

To further clarify whether the Maresin1–LGR6 axis directly regulates the transcriptional activity of mitochondrial biogenesis-related genes, we performed a dual-luciferase reporter assay (Supplementary Fig. 13a). Under HS conditions, cells were treated with Maresin1 (50 or 100 nM) in combination with LGR6 knockdown via siRNA (siRNA targeting LGR6 (siLGR6) or a negative control (siNC)) to evaluate its regulatory effects. The results showed that in the siNC control group, Maresin1 significantly enhanced PGC1α promoter activity, whereas this enhancing effect was markedly attenuated or abolished under siLGR6 conditions (Supplementary Fig. 13b).

### Deletion of LGR6 in cardiomyocytes leads to mitochondrial dysfunction in heatstroke

**Fig. 8:**
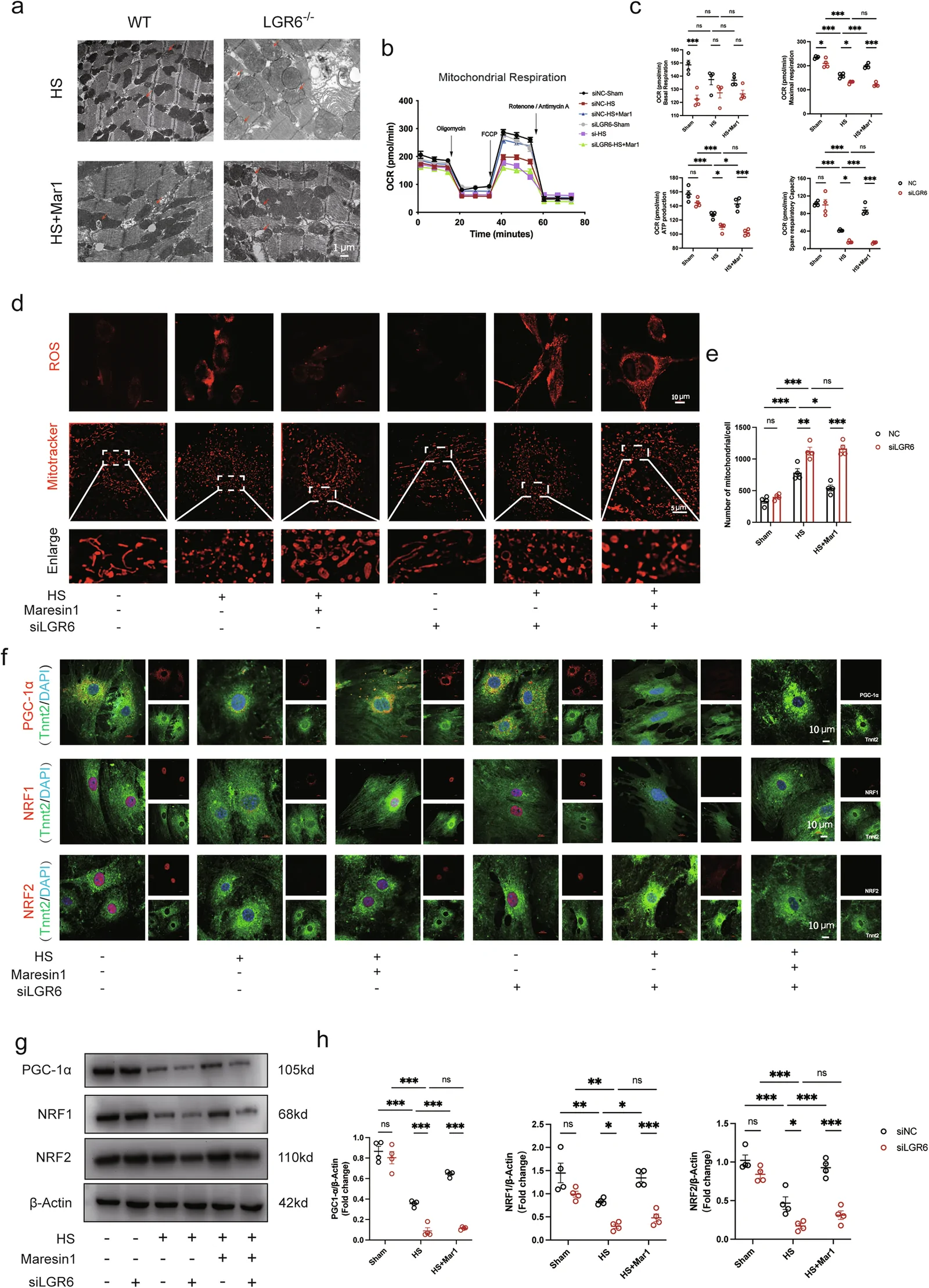
Deletion of LGR6 in cardiomyocytes leads to mitochondrial dysfunction. **a** TEM images of ventricular tissues from WT and *Lgr6*^−/−^ mice (scale bar = 1 μm). **b**,**c** Real-time monitoring of mitochondrial OCR in siNC and siLGR6 cardiomyocytes. Left: OCR diagram. Right: Quantification of maximal respiration, basal respiration, ATP production, and spare mitochondrial capacity. **d**,**e** Mitochondrial ROS levels in cardiomyocytes stained with MitoSOX Red, assessed via immunofluorescence (scale bar = 10 μm). Representative images and analysis of MitoTracker Red staining in siNC and siLGR6 cardiomyocytes (scale bar = 20 μm; *n* = 4). **f** Immunofluorescence staining of PGC1α, NRF1, and NRF2 (red), Tnnt2 (green), and DAPI (blue) in siNC and siLGR6 cardiomyocytes (scale bar = 20 μm). **g**,**h** Western blot analysis and quantification of PGC1α, NRF1, and NRF2 in cardiomyocytes across all groups (*n* = 4). Statistical analyses were conducted using two-way ANOVA, followed by Tukey’s multiple comparisons test (**c**,**e**,**h**). Data are expressed as mean ± s.e.m.. Statistical significance is indicated as follows: ns, no significant difference; *P < 0.05, **P < 0.01, ***P < 0.001.

HS-induced cardiac dysfunction is primarily characterized by mitochondrial damage in cardiomyocytes. Maresin1 primarily confers cardioprotective effects through the LGR6 receptor on cardiomyocytes. To explore the role of LGR6 in Maresin1-mediated protection against HS-induced cardiomyocyte damage in vivo, TEM revealed increased mitochondrial swelling and rupture, disorganized myofibrils, and significant cytoplasmic vacuolation in *Lgr6*-knockout mice (Fig. 8a). Furthermore, ATP content measurements in cardiac tissue showed a significant reduction in both HS and HS+Mar1 groups of *Lgr6*-knockout mice, further supporting the importance of LGR6 in preserving mitochondrial function (Supplementary Fig. 5). In vitro, cardiomyocytes were transfected with siLGR6 or siNC. Cells were then subjected to sham, HS, or HS+Mar1 treatments (the silencing efficiency of siLGR6 is shown in Supplementary Fig. 6). Mitochondrial respiration was evaluated with the Seahorse XF-96 extracellular flux analyzer. Interestingly, siLGR6 transfection under sham conditions did not significantly alter the basal expression of PGC1α, NRF1, or NRF2. Under HS conditions, the loss of LGR6 markedly suppressed these mitochondrial biogenesis regulators, highlighting a stress-dependent activation requirement of the LGR6 signaling axis (Fig. 8b,c). In vivo immunoblot analysis of cardiac tissues from AAV-null and AAV-LGR6 mice revealed that cardiomyocyte-specific knockdown of LGR6 significantly reduced the expression of mitochondrial biogenesis-related proteins (Supplementary Fig. 8) and Electron Transport Chain (ETC) complex proteins (Supplementary Fig. 11), while increasing the expression of mitochondrial damage-associated genes (*Dloop, mt-Nd1, mt-Nd2*) under HS conditions compared with the AAV-null group (Supplementary Fig. 12). To investigate the mechanisms involved, immunofluorescence staining and western blot analyses were conducted to assess mitochondrial morphology, oxidative stress, and key regulators of mitochondrial function. Cardiomyocytes exposed to HS and transfected with siLGR6 exhibited significantly increased ROS levels and severe mitochondrial damage, characterized by fragmented and swollen networks, as observed through MitoTracker staining. These mitochondrial issues were more pronounced in siLGR6-transfected cells than in WT controls, regardless of Maresin1 treatment (Fig. 8d,e). Considering the importance of mitochondrial biogenesis in ATP production and cardiac function, we investigated the impact of the Maresin1–LGR6 axis on mitochondrial biogenesis. Under HS conditions, siLGR6-transfected cardiomyocytes displayed reduced expression of mitochondrial biogenesis-related proteins and genes (PGC1α, NRF1, and NRF2), regardless of Maresin1 treatment (Fig. 8f–h and Supplementary Fig. 10). Simultaneously, we analyzed the expression of genes associated with mitochondrial biogenesis, stress response, apoptosis, and mitochondrial dynamics after siNC or siLGR6 treatment (Supplementary Fig. 14). The results showed that LGR6 knockdown significantly affected the mitochondrial biogenesis-related gene network, with marked changes observed in the expression of *Tfam* and *Polg*. In contrast, no significant differences were detected in mitochondrial stress response markers (*Atf4*, *Ddit3/Chop*), apoptosis-related genes (*Bax*, *Bcl2*), or mitochondrial dynamics regulators (*Dnm1l/Drp1*, *Mfn1*). These results underscore the critical role of LGR6 in mediating the cardioprotective effects of Maresin1 against HS-induced damage.

## Discussion

**Fig. 9:**
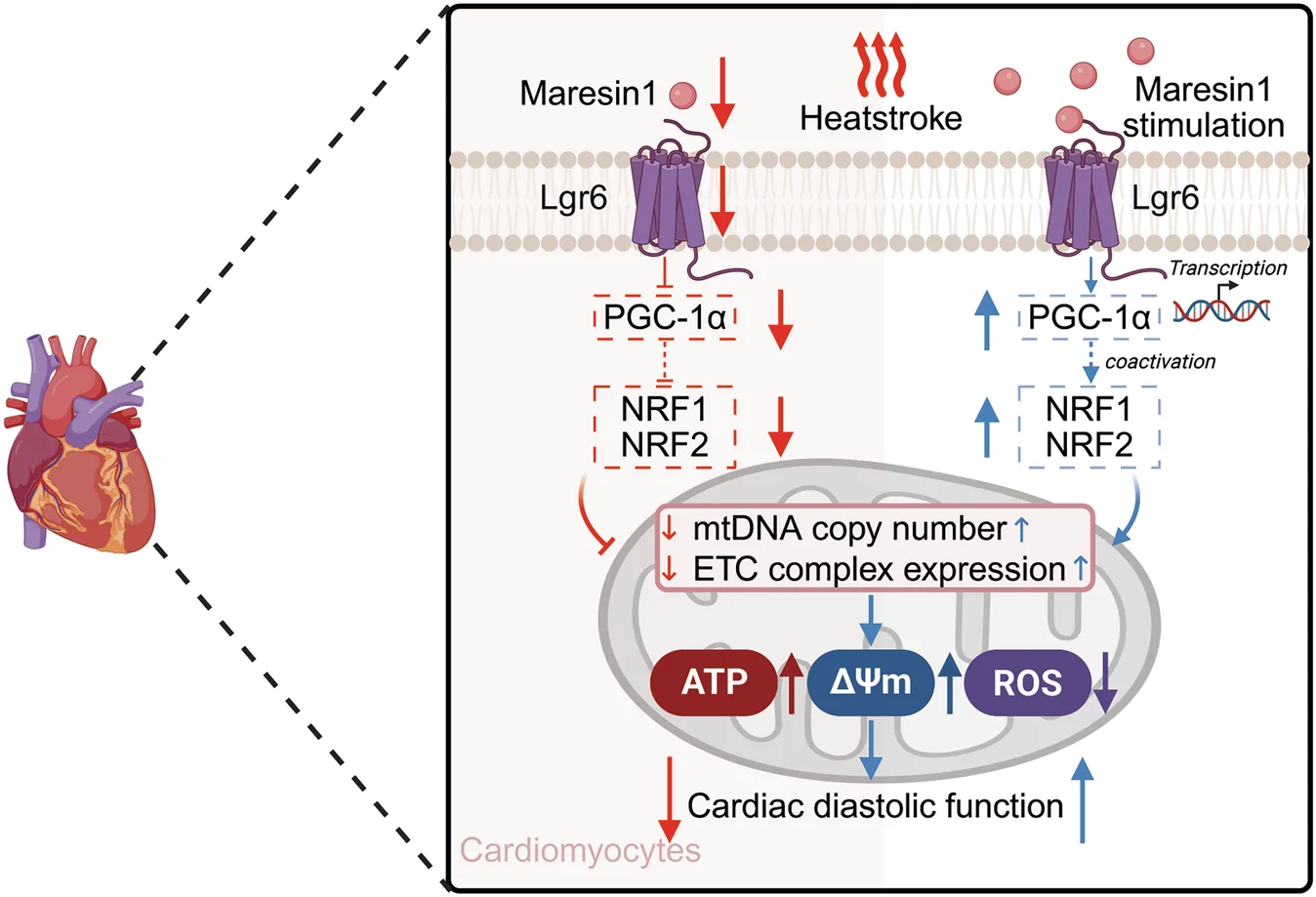
Schematic diagram of the cardioprotective mechanism of the Maresin1/LGR6 axis in heatstroke. Heatstroke decreases endogenous Maresin1, thereby suppressing the LGR6/PGC-1α/NRF1/NRF2 signaling axis. This suppression disrupts mitochondrial function, causing ROS overproduction, reduced ATP synthesis, and loss of membrane potential (ΔΨm), which collectively drive cardiac dysfunction. Maresin1 administration activates the LGR6 receptor to reactivate this pathway and preserve mitochondrial integrity. Red arrows denote heatstroke-induced impairments; blue arrows indicate the protective eff ects of Maresin1. ΔΨm: mitochondrial membrane potential; ETC: electron transport chain.

In this study, we observed the downregulation of Maresin1 expression in both the plasma and cardiac tissue of HS mice, with an upregulation of LGR6 receptor expression in cardiomyocytes. Maresin1, a pro-resolving lipid mediator with anti-inflammatory properties, may play a crucial role in resolving inflammation and mitigating worsened tissue damage. The upregulation of LGR6 could represent a compensatory mechanism to enhance Maresin1 signaling by increasing receptor expression, thereby promoting protective pathways such as AKT and Wnt/β-catenin. Exogenous Maresin1 treatment alleviated myocardial damage and improved diastolic function in HS mice, whereas LGR6 deficiency, regardless of Maresin1 treatment, led to adverse effects. Transcriptomics analysis revealed that oxidative stress occurs in myocardial cells during HS, and Maresin1 mitigates this damage, as shown by electron microscopy and immunofluorescence. Using *Lgr6*-knockout mice and LGR6 siRNA, we investigated the Maresin1–LGR6 axis in HS. Maresin1 reduced oxidative stress, improved mitochondrial function via the LGR6 receptor, increased mitochondrial protein synthesis (PGC1α, NRF1, and NRF2), enhanced ATP production, and decreased oxygen consumption. Specifically, Maresin1 significantly increased PGC1α expression and promoter activity in an LGR6-dependent manner, as demonstrated by luciferase reporter assays. Importantly, NRF1 and NRF2 expression levels were also elevated after Maresin1 treatment; however, these changes appeared to occur downstream of PGC1α activation. Therefore, these compelling findings establish a prominent role of Maresin1 in HS, as illustrated in Fig. 9.

Maresin1, a specialized pro-resolving lipid mediator produced by macrophages during inflammation resolution, is key in reducing inflammation and supporting tissue repair^11,13^. The serum and cardiac tissue levels of Maresin1 were downregulated after HS, similar to those observed in patients with active rheumatoid arthritis, obesity, and type 2 diabetes^27,28^. Compared with healthy individuals, plasma MaR1 concentrations were reduced in patients with active rheumatoid arthritis^29^. Serum MaR1 levels are markedly reduced in patients with type 2 diabetes, especially those with diabetic foot ulcers or nephropathy^30^. These results suggest that a decreased Maresin1 level may be a warning sign for HS.

Maresin1 influences signaling pathways by binding to receptors like LGR6, a G-protein-coupled receptor with leucine-rich repeats, to suppress inflammation, promote cellular debris clearance, and aid in tissue regeneration^31^. In our data, flow cytometry and immunofluorescence analysis of cardiac tissue revealed that LGR6 is elevated after HS, a finding similar to those observed in diabetic cardiomyopathy. The upregulation of LGR6 may activate downstream signaling pathways crucial in tissue repair, stem cell maintenance, and cellular metabolism. The involvement of LGR family in cardiovascular diseases has garnered significant attention in recent years^32^. Studies have reported that overexpressing LGR6 in cardiomyocytes can regulate mitochondrial biogenesis and ferroptosis, potentially treating diabetic cardiomyopathy^21^.

In addition, LGR6 reportedly inhibits necroptosis in cardiomyocytes, thereby protecting against myocardial ischemia–reperfusion injury^20^. Beyond its cardioprotective properties, LGR6 plays a significant role in pulmonary hypertension, abdominal aortic aneurysm, osteoporosis, and lung inflammation^15,23,31,33^. Furthermore, using *Lgr6*^−/−^ mice, we observed that the protective effects of Maresin1 on cardiac function and its inhibition of mitochondrial oxidative stress were abolished.

In recent years, mitochondrial oxidative stress has become a key focus in studying the pathophysiology of HS-induced cardiac damage^8^. In some in vitro models of HS-induced cardiomyocyte injury, studies have found that reducing mitochondrial ROS accumulation can improve vascular endothelial cell function in acute lung injury and mitigate HS-induced myocardial injury^34,35^, underscoring the importance of targeting mitochondrial pathways to mitigate cardiomyocyte injury and improve cardiac outcomes^7,8,35,36^. PGC1α plays a crucial role in cardiac energy metabolism. Knocking down PGC1α in cardiomyocytes reportedly disrupts cardiac metabolism, impairing cardiac function^37^. Our study suggests that exogenous Maresin1 administration in the HS model mitigated mitochondrial oxidative stress and enhanced the expression of mitochondrial PGC1α, NRF1, and NRF2 proteins. However, cardiomyocytes still exhibited oxidative stress damage after silencing LGR6, suggesting a potential relationship between the Maresin1–LGR6 axis and mitochondrial oxidative stress damage.

Our findings reveal a profound context-dependent role for LGR6 in cardiomyocyte survival and mitochondrial function. Under sham conditions, constitutive LGR6 expression appears to play a limited or redundant role, as LGR6 ablation did not perturb basal cardiac function or the baseline expression of mitochondrial biogenesis factors (PGC1α, NRF1, and NRF2). However, under HS, this dynamic changes drastically. The upregulation of LGR6 after HS likely represents a compensatory ‘stress response’ mechanism aimed at maximizing the capture of available pro-resolving ligands like Maresin1. Furthermore, the striking divergence in the phenotypic consequences of LGR6 deletion between sham and HS conditions suggests an altered sensitivity of its downstream signaling pathways under stress. During severe hyperthermia and oxidative stress, basal regulatory mechanisms are overwhelmed, rendering cardiomyocytes entirely dependent on the Maresin1–LGR6 axis to sustain PGC1α/NRF-mediated mitochondrial biogenesis. This stress-dependent activation paradigm explains why exogenous Maresin1 exerts such profound protective effects precisely when LGR6 signaling is sensitized by the HS environment.

Despite our findings, the study has limitations, notably its reliance on a mouse model, for which the clinical relevance remains to be confirmed. Human translational studies are needed to validate these findings. In addition, we used *Lgr6*^−/−^ mice to systematically knock out LGR6 and explore the role of the Maresin1–LGR6 axis in HS-induced cardiac injury. Using mice with cardiomyocyte-specific *Lgr6*-knockout could offer a more precise insight into its function within cardiomyocytes.

## Supplementary information

Supplementary Information
