# Supplementary material for: Maresin1 improves cardiac dysfunction via LGR6 in heatstroke
Source: Exp Mol Med. 2026 Oct 2;58(10):3395–408. doi: 10.1038/s12276-026-01853-6 (PMC13736894; doi:10.1038/s12276-026-01853-6)
Supplement: Supplementary file 1 [file 12276_2026_1853_MOESM1_ESM.docx]

**Supplemental Materials**

**1.1 Animal preparation**

ARRIVE guidelines guided all the animal experiments, and the procedures were approved by the Wenzhou Medical University Animal Ethics Committee (Approval No. wydy2025-0104) and coincided with the Guide for the Care and Use of Laboratory Animals issued by the National Institutes of Health. 120 Male C57BL/6J wild-type mice, aged 6–8 weeks and weighing 20–25 g, were sourced from Charles River Lab China. LGR6 knockout (LGR6^-/-^) mice were obtained from the Shanghai Model Organisms Centre, Inc., with genotyping confirmed through genomic PCR (Fig.S1). All animals had unrestricted access to food and water, and the experimental protocols were approved by the Institutional Animal Care and Use Committee (IACUC). Our randomization method was as follows: each animal was assigned a temporary number with a marker, and the cages were randomly numbered. Using an online random number generator, the mice were randomly assigned to the randomly numbered cages. Only the investigator Yu-jie Zhou and Li-ran Su administered the intervention based on the randomization table and aware of the treatment group allocation. The animal exclusion criteria are as follows: we exclude animals that experience significant weight loss (a rapid decrease of 15-20% compared to their initial weight), weakness (unable to eat or drink), or dull fur. Researchers should visit the animals at least twice a day to ensure they have adequate food and water supplies, observe their condition, and if necessary, provide individual cage feeding. When handling the animals, care should be taken to move them gently and alleviate fear and agitation by touching the animals.

**1.2 LC-MS/MS**

Plasma Maresin1 levels were quantified using liquid chromatography-tandem mass spectrometry (LC-MS/MS) based on established protocols, with Maresin1 included as an internal standard in each sample. The extraction process involved ethyl acetate (4:1 vol/vol, ethyl acetate to sample ratio) and vortexing to precipitate proteins and centrifugation. The resulting supernatants were dried under nitrogen gas and reconstituted in 100 µL of methanol. Calibration curves were prepared using a plasma substitute of methanol and water (1:1 vol/vol) with analyte concentrations ranging from 0.5 to 200 pg/mL, showing excellent linearity (r² = 0.98–0.99). Chromatographic separation was performed on an Agilent Eclipse Plus C18 column (2.1 mm × 100 mm × 1.7 µm; Agilent, Santa Clara, CA, USA). Mass spectrometric analysis was conducted using a Sciex 6500 QTRAP mass spectrometer (Sciex, Redwood City, CA, USA). Instrument control and data acquisition were managed with Analyst™ 1.6.3 software (Applied Biosystems, Foster City, CA, USA). Mice in this part were finally divided into 2 groups: Sham (The environment was set at 37°C with 40 ± 5 % relative humidity for 2 hours, n=5), HS group(After 2 hours in a 39°C with 60 ± 5 % relative humidity environment, the mice were transferred to normal conditions for 12 hours, n=5).

**1.3 RNA-seq analysis**

Total RNA was extracted from ventricular tissues of wild-type and heatstroke mice, both with and without Maresin1 treatment, with three biological replicates per group. Apexbio (Shanghai, China) conducted RNA-seq experiments. Total RNA extraction was performed using TRIzol, and differential expression analysis was carried out using the R package (version 3.12.1). Using the Benjamini–Hochberg method, P values were adjusted to control the false discovery rate, with genes showing an adjusted P value < 0.05 considered differentially expressed. Hierarchical clustering heatmaps were generated using the gplot library. The cluster Profiler R package performed the Gene Ontology (GO) and Kyoto Encyclopaedia of Genes and Genomes (KEGG) pathway enrichment analyses for differentially expressed genes, accounting for gene length bias. GO terms and KEGG pathways with a corrected P value < 0.05 were deemed significantly enriched. Mice in this part were finally divided into 3 groups: Sham (n=3), HS group(n=3), HS+Maresin1 group (Mice were placed in an environment at 39°C with 60 ± 5% relative humidity for 2 hours, and then immediately received a tail vein injection of Maresin1, n=3).

**1.4 Transthoracic Echocardiography**

Mice underwent trans-thoracic echocardiography using the Vevo 3100 imaging system. Anaesthesia was induced by placing the mice in an anaesthesia chamber, and anaesthetised mice were positioned supine on a heating pad. The isoflurane flow rate was maintained at 0.6–1 L/min to achieve a target heart rate of 470 ± 50 bpm. To ensure accurate experimental data, the depth of anaesthesia was carefully controlled, as tighter heart rate ranges yielded more accurate readings. Hair removal was conducted a day before the procedure. Conductive adhesive was applied to electrodes, and the mice were securely positioned on a small animal operating table. Ultrasonography gel was applied to the thoracic and upper abdominal region to facilitate imaging.

Respiratory rates were continuously monitored during imaging to ensure physiological stability. M-mode images were captured from long- and short-axis views to assess left ventricular ejection fraction (EF) and fractional shortening (FS). Left ventricular diastolic function was evaluated using tissue Doppler imaging (TDI) and pulse wave Doppler (PWD) modes from the apical four-chamber view. PWD quantified regional haemodynamics, while TDI measured myocardial blood flow velocity. Colour Doppler mode visualised blood flow and parameters such as aortic and pulmonary artery blood flow were calculated using the B-mode from various positions. Each parameter was measured at least three times, and the mean values were used for analysis.

All mice recovered from anaesthesia without complications upon completing the procedures. Finally, mice in the experiment were divided into 5 groups: WT Sham group(n=11), WT HS group(n=16), WT HS+Maresin1 group(n=11), LGR6^-/-^ HS group(n=5), LGR6^-/-^ HS+Maresin1 group(n=5).

**1.5 Enzyme-Linked Immunosorbent Assay (ELISA) and Biochemical Estimation**

Plasma samples from mice were analysed using ELISA kits for cardiac troponin I (cTnI, JL40538, Jianglaibio, Shanghai) and N-terminal pro-B-type natriuretic peptide (NT-proBNP, JL11641, Jianglaibio, Shanghai). Plasma levels of alanine aminotransferase (ALT), aspartate transaminase (AST), creatine kinase (CK), creatine kinase isoenzymes (CK-MB), lactate dehydrogenase (LDH), and lactate dehydrogenase isoenzymes (LDH1) were measured using an AU480 auto-analyser (Beckman Coulter). Mice in this part were finally divided into 5 groups: WT Sham group(n=5), WT HS group(n=10), WT HS+Maresin1 group(n=10), LGR6^-/-^ HS group(n=5), LGR6^-/-^ HS+Maresin1 group(n=5). In Figure 1C-E, the HS group was further subdivided into HS groups with normal feeding for 0, 6, and 12 hours(n=15).

**1.6 Heart histology and immunofluorescence assay**

Mice were anaesthetised with 1% pentobarbital (50 mg/kg). Following perfusion with PBS through the right ventricle, the hearts were excised and fixed in 4% paraformaldehyde overnight at 4°C. The samples were dehydrated, embedded in paraffin, and sectioned into 6μm slices. These sections were mounted on poly-lysine-coated slides and stained with haematoxylin and eosin (H&E).

Primary mouse cardiomyocytes were plated on confocal dishes (NEST, 801002, China) the evening before immunofluorescence analysis. Both cardiomyocytes and tissue sections were permeabilised with 0.2% Triton X-100 and blocked with 5% bovine serum albumin (BSA). Samples were incubated overnight at 4°C with primary antibodies targeting peroxisome proliferator-activated receptor-γ co-activator-1α (PGC-1α), nuclear respiratory factor 1 (NRF1), and nuclear respiratory factor 2 (NRF2), co-stained with an antibody against cardiac troponin T (Tnnt2, MA5-12960, Invitrogen). After three PBS washes, sections were incubated for an hour at normal room temperature with Alexa Fluor-conjugated secondary antibodies specific to the primary antibodies. Nuclei were counterstained with DAPI.

All steps were conducted under light-protected conditions. Images were captured using a confocal microscope, and all experiments included at least three biological replicates. In this experiment, only mouse plasma was used. The remaining heart tissue was embedded in paraffin to make tissue sections for subsequent HE staining and immunofluorescence staining. Part of the tissues were homogenized to extract RNA for qPCR, part were used to extract protein for WB experiments, and part were used to measure the ATP and MDA content in the heart tissue.

**1.7 Transmission electron microscopy**

After anaesthetising the mice, heart tissues were collected and immersed in PBS. Ventricular tissues were cut into 2mm³ cubes (2 × 1 × 1 mm) and fixed in 2.5% glutaraldehyde at room temperature under light-protected conditions for 2 days. The fixed tissue blocks were then embedded in epoxy resin. Ultrathin sections (50–70 nm) were prepared from the resin blocks and examined using a Hitachi H-7650 electron microscope (Hitachi, H-7650, Tokyo, Japan). Mice in the experiment were divided into 3 groups: Sham group (n=3), HS group (n=3), HS+Maresin1 group (n=3).

**1.8 Quantitative real-time polymerase chain reaction analysis**

Total RNA was extracted by homogenising 30 mg of left ventricular tissue in 1 ml of RNiso Plus (9108Q, Takara, China) in a 1.5 ml RNase-free EP tube, following the manufacturer's instructions. For cultured cells in 35 mm dishes, RNA was extracted using 0.5 ml of RNiso Plus, and a NanoDrop 1000 spectrophotometer (Thermo Fisher Scientific, USA) measured the RNA Concentrations. cDNA synthesis was performed using PrimeScript™ RT Master Mix (RR047Q, Takara, China). Quantitative real-time PCR (qRT-PCR) was conducted with TB Green Premix Ex Taq™ II (RR420Q, Takara, China), ensuring at least one technical replicate per sample. Gene expression levels were analysed using the 2-ΔΔCt method. Primer sequences are listed in Supplementary Table 1.

**1.9 Flow cytometry of mouse heart tissue**

Mice were anaesthetised via intraperitoneal injection of sodium pentobarbital. A digestion enzyme solution was prepared by mixing 0.25 mg/ml Liberase TL (5401020001, Sigma-Aldrich) and 20 μg/ml DNase I (10104159001, National Pharmaceutical Chemical Reagent) in PBS. Peripheral blood was flushed from the heart chambers by injecting 20 ml of PBS into the right ventricle. The thymus was lifted to expose the heart, which was then excised and immersed in sterile PBS. Ventricular tissue was sectioned into approximately 1mm³ cubes and placed into a 1.5 ml EP tube containing the digestion enzyme solution, incubated at 37°C for 15 minutes.

The digestion process was halted using flow cytometry buffer (PBS supplemented with 10% FBS and 5% EDTA). A 100μm strainer filtered the cell suspension to remove undigested tissue fragments, washed, and resuspended in 100 μl of flow cytometry buffer. Cells were blocked for 5 minutes, followed by staining with monoclonal antibodies at 4°C in the dark for 30 minutes. After staining, cells were washed twice at 300g for 5 minutes, and the resulting cell pellet was resuspended in 300 μl of flow cytometry buffer to create a single-cell suspension for downstream experiments.

The gating strategy was based on Reference [30]: cardiomyocytes were gated as Tnnt2+, immune cells as CD45+, endothelial cells as CD31+, and fibrocytes as CD90+. All cell types were further gated for LGR6 expression. Flow cytometric analysis was performed using a BD Canto II. A complete list of antibodies is provided in Supplementary Table 2. Mice in the experiment were divided into 2 groups: Sham group (n=5), HS group (n=5).

**1.10 Detection of ATP Content in Cardiac Tissue and Cardiomyocytes**

All procedures followed the ATP Assay Kit instructions (S0027, Beyotime). ATP standard solutions with concentrations of 0.01, 0.03, 0.1, 0.3, 1, 3, and 10 µM were prepared to generate a standard curve.

For cardiac tissue, 20 mg of heart tissue was weighed, and 100 µl of lysis buffer was added. The tissue was homogenised and centrifuged at 12,000g for 5 minutes at 4°C to collect the supernatant. Cells were further seeded in 35 mm dishes for cardiomyocytes and incubated overnight to achieve 90% adherence. After the designated treatment conditions, 200 µl of lysis buffer was added to each dish to ensure complete lysis. The lysates were centrifuged at 12,000g for 5 minutes at 4°C, and the supernatant was collected.

The ATP detection solution was prepared according to the kit's instructions. Each well received 100 µl of the working solution and was incubated at room temperature for 5 minutes. Subsequently, 20 µl of either the standard solution or the sample was added to the wells mixed thoroughly, and the luminescence intensity (RLU) was measured using a chemiluminescence analyser. The ATP concentration was determined by comparing the RLU values to the standard curve.

**1.11 MDA content detection**

Cardiac tissue was lysed using cell lysis buffer (Beyotime, P0013), and the 4°C homogenates were centrifuged for 10 mins at 12,000g to collect the supernatant for malondialdehyde (MDA) analysis. The MDA detection working solution was prepared following the instructions provided with the assay kit (Beyotime, S0131S).

A standard curve measuring absorbance at 532 nm was constructed by preparing a series of MDA standard solutions with varying concentrations. Supernatant samples were then mixed with the MDA detection working solution incubated according to the protocol to form MDA-TBA adducts, and their absorbance was measured at 532 nm.

The MDA concentrations in the samples were calculated based on the standard curve, providing a reliable assessment of lipid peroxidation and oxidative stress in cardiac tissue.

**1.12 Assessment of Reactive Oxygen Species (ROS) and Mitochondrial Membrane Potential**

Primary cardiomyocytes were seeded into 35 mm confocal dishes and allowed to adhere overnight. Detection solutions were prepared using a culture medium containing 5µM JC-1 (C2006, Beyotime) or MitoSOX Red (S0061S, Beyotime). Cells were incubated with these detection solutions at 37°C for 30 minutes and washed thrice with PBS.

A confocal microscope equipped with a live-cell workstation was used for imaging analysis. For flow cytometry analysis (Beckman Coulter, CytoFLEX), the cells were gently detached from the culture dishes using a cell scraper to generate a single-cell suspension in the culture medium. The cells were then stained with the exact detection solutions and analysed for mitochondrial membrane potential and ROS levels.

**1.13 Mitochondrial stress analysis**

The oxygen consumption rate (OCR) was measured in real time using the XFe96 extracellular flux analyser. Cardiomyocytes were seeded at a density of 5 × 10⁴ cells per well in XFe96 cell culture microplates and incubated overnight at 37°C. Concurrently, the sensor plate was hydrated overnight in a non-CO₂ incubator at 37°C.

The following day, the Seahorse assay medium was prepared (containing 10 mM glucose, 10 mM pyruvate, and 10 mM glutamine, adjusted to pH 7.4). The cells were washed twice with the assay medium, leaving 180 µl in each well upon removing the culture medium from the wells. The sensor plate was further hydrated with an XF calibration solution for 1 hour. Stock and working solutions of mitochondrial respiration-modulating or inhibiting compounds were prepared and sequentially added to the assay cartridge.

First, the "basal OCR" was recorded, representing cellular respiration under standard physiological conditions. Oligomycin (1.5 μM) was then introduced to evaluate mitochondrial "proton leak" and "ATP-linked OCR." Next, the uncoupling agent carbonyl cyanide-p-trifluoromethoxy phenylhydrazone (FCCP; 3 μM) was added to measure the maximum respiratory rate, with the difference between this rate and the basal OCR reflecting the "reserve capacity" of the electron transport chain. To account for non-mitochondrial contributions to OCR, a combination of rotenone (0.5 μM) and antimycin-A (0.5 μM) was used to inhibit Complexes I and III, respectively, enabling the measurement of non-mitochondrial OCR ("NM OCR").

**1.14 siRNA transfection**

Cardiomyocytes were transfected with either negative control small interfering RNA (siRNA, siNeg) or siRNAs targeting LGR6, following the manufacturer's instructions (Gene Pharma, Shanghai, China). The transfection process utilised Lipofectamine™ 3000 Transfection Reagent (Thermo Fisher Scientific, L3000008). To confirm the efficiency of siRNA transfection, total protein was extracted and analysed using a western blot. The primer sequences used included a non-targeting control siRNA (siNC) with the sense sequence 5′-UUCUCCGAACGUGUCACGUTT-3′ and an LGR6-specific siRNA (siLGR6) with the sense sequence 5′-GCGCCUACAUCAAGCUCUAT-3′.

**1.15 Western blot analysis**

Approximately 30 mg of ventricular tissue was placed in a 1.5 ml EP tube, and lysis buffer (RIPA: PMSF = 99:1, Solarbio, China) was added. The tissue was homogenised for 2 minutes and subjected to ultrasonication at 4°C for three 5-minute cycles. The 4°C lysate was centrifuged for 15 mins at 12,000g, and the supernatant was collected. Protein concentrations were measured, and samples were mixed with loading buffer and heated at 95°C for 10 minutes.

Proteins were separated using SDS-polyacrylamide gel electrophoresis and transferred onto PVDF membranes. The membranes were blocked with NcmBlot blocking solution (New Cell & Mechanical Biotech, P30500) for 15 minutes and incubated with primary antibodies (1:1000 dilution) at 4°C for 12–16 hours. Afterwards, membranes were incubated with secondary antibodies (1:3000 dilution) for 2 hours at normal room temperature.

Protein bands were visualised using a chemiluminescence substrate and a UVPFA Gel Imaging System (Upland, California). Band intensities were quantified using ImageJ software. A complete list of antibodies is provided in Supplementary Table 2.

**1.16 In vivo observation with thermal imaging**

For thermal imaging analysis, the IRTool Pro system was employed to record the body temperature of mice following Sham and Heatstroke mice. After establishing the HS model, mice from both the HS and Sham groups were anesthetized with isoflurane, and their limbs were fixed. The IRTool Pro system was then used to monitor their whole-body temperature.

**1.17 Construction of adeno-associated virus for cardiomyocyte specific LGR6 knockdown in mice.**

Based on the Lgr6 gene (Gene ID: 329252), shRNA interference plasmids were constructed using the miR30 structure. The plasmids were designed to express shRNA and the EGFP reporter gene, driven by the cardiomyocyte-specific cTNT promoter. The experimental plasmids included pAAV-cTNT>Kozak-EGFP/mLgr6[miR30-shRNA], while the control plasmid contained only cTNT>Kozak-EGFP. The correctly constructed plasmids were then packaged into AAV9 serotype viruses. To ensure efficient and specific infection and gene knockdown in cardiomyocytes, the viral titer was ≥1×10¹³ vg/mL. The constructed viruses were injected into WT mice via tail vein and administered continuously for one week. Subsequent experiments were conducted three weeks after the final injection.

**1.18 Double luciferase reporting assay**

To assess the transcriptional activity of PGC-1α, NRF1, and NRF2 promoters, cells were transfected with luciferase reporter constructs containing the corresponding promoter sequences or an empty pGL3-basic vector (negative control), together with the reference plasmid pRL-TK for normalization. To investigate the regulatory effect of LGR6–Maresin1 signaling on promoter activity, cells were transfected with siLGR6 or siNC, followed by treatment with Maresin1 (50 or 100 nM) under both sham and HS conditions. The transcriptional activity of each promoter construct was then evaluated using a Dual Luciferase Reporter Gene Assay Kit (Vazyme, DL101) according to the manufacturer’s instructions. All luciferase activities were normalized to Renilla luciferase activity, and experiments were independently performed at least four times. The promoter reporter plasmids for PGC-1α, NRF1, and NRF2, as well as the empty vector control, were obtained from OBiO Technology (Shanghai, China).

**Supplemental Figures**

**Supplementary Fig. 1: Global LGR6**^−/−^ **mice were used in this study.**

(a-b) Left: Genotypes were examined with four different primers. Right: Protocol diagram for the study design. (c-d) Western blot and quantification of LGR6 expression in heart tissues from WT and LGR6^−/−^ mice(n=9). Mean ± SEM. d shows a two-tailed unpaired Student's t-test analysed the data. Statistical significance is indicated as follows: ns, no significant difference; **p* < 0.05; ***p* < 0.01; ****p* < 0.001.

**Supplementary Fig. 2:** **Plasma Biochemical Analysis in WT with and without Maresin1 Treatment under Heatstroke.**

Plasma concentrations of AST, ALT, LDH1 and CK-MB were measured in WT subjected to heatstroke (HS) with or without Maresin1(Mar1) treatment (n=5 per group). Data were analysed separately for each treatment condition to evaluate the biochemical impact of Maresin1(n=5). Mean ± SEM shows the data. a-d were analysed using one-way ANOVA and Tukey's multiple comparisons test. Statistical significance is indicated as follows: ns, no significant difference; **p* < 0.05; ***p* < 0.01; ****p* < 0.001.

**Supplementary Fig. 3:** **Plasma Biochemical Analysis in WT and LGR6 Knockout Mice with and without Maresin1 Treatment in Heatstroke.**

Plasma concentrations of AST, ALT, LDH1 and CK-MB were measured in WT and LGR6 knockout (LGR6^-/-^) mice subjected to heatstroke (HS) with or without Maresin1(Mar1) treatment (n=5 per group). Data were analysed separately for each treatment condition to evaluate the biochemical impact of Maresin1(n=5). Data are presented as mean ± SEM. a-d were analysed using two-way ANOVA and Tukey's multiple comparisons test. Statistical significance is indicated as follows: ns, no significant difference; **p* < 0.05; ***p* < 0.01; ****p* < 0.001.

**Supplementary Fig. 4:** **Heatstroke-induced cardiomyocyte injury model.**

(a) Cardiomyocytes were exposed to 39°C at different times, and Nppa expression was analysed using qPCR(n=5). (b) After 2 hours of exposure to 39°C, cardiomyocytes were treated with different doses of Maresin1 for 12 hours, then change the medium. Nppa expression was measured via qPCR 48 hours post-treatment(n=5). Mean ± SEM shows the data. A and B were analysed using two-way ANOVA and Tukey's multiple comparison tests. Statistical significance is indicated as follows: ns, no significant difference; **p* < 0.05; ***p* < 0.01; ****p* < 0.001.

**Supplementary Fig. 5: Cardiac ATP Levels in WT and LGR6**^−/−^ **Mice Under Heatstroke with or without Maresin1 Treatment.**

The ATP concentrations in cardiac tissue were assessed in WT and LGR6^−/−^ mice subjected to heatstroke (HS), with or without Maresin1 treatment. The study found a notable decrease in ATP levels in LGR6^−/−^ mice relative to WT, underscoring LGR6's importance in sustaining mitochondrial energy production during HS and the cardioprotective benefits of Maresin1. Mean ± SEM shows data. A and B were analysed using two-way ANOVA and Tukey's multiple comparisons test. Statistical significance is indicated as follows: ns, no significant difference; **p* < 0.05; ***p* < 0.01; ****p* < 0.001.

**Supplementary Fig. 6: The silencing efficiency of siLGR6 in cardiomyocytes.**

Cardiomyocytes were transfected with small interfering a negative control (siNC) or RNA (siRNA) targeting LGR6 (siLGR6). (a-b) Western blot analysis and quantification of Lgr6 expression cardiomyocytes(n=6). Data are presented as mean ± SEM and analysed using a two-tailed unpaired Student's t-test. Statistical significance is indicated as follows: ns, no significant difference; **p* < 0.05; ***p* < 0.01; ****p* < 0.001.

**Supplementary Fig. 7: Infrared thermography results showing core body temperature in Sham and HS groups.**

Representative infrared thermal images of mice in Sham and HS groups, illustrating the temperature in the cardiac region of the mice. The HS group shows a significant increase in core body temperature compared to the Sham group (n=5). Data are presented as mean ± SEM and analysed using a two-tailed unpaired Student's t-test. Statistical significance is indicated as follows: ns, no significant difference; **p* < 0.05; ***p* < 0.01; ****p* < 0.001.

**Supplementary Fig. 8: Cardiomyocyte-specific LGR6 knockdown suppresses the expression of mitochondrial function-related proteins.**

Immunoblot analysis and statistical results of the levels of mitochondrial function-related proteins (NRF1, NRF2 and PGC-1α) in cardiac tissues from cardiomyocyte-specific LGR6 knockdown (AAV-LGR6) and AAV-control (AAV-null) mice (n=3). Data are presented as mean ± SEM and analysed using two-way ANOVA and Tukey's multiple comparison tests. Statistical significance is indicated as follows: ns, no significant difference; **p* < 0.05; ***p* < 0.01; ****p* < 0.001.

**Supplementary Fig. 9: Expression of LGR6, PGC-1α, NRF1 and NRF2 in cardiac tissue following Maresin1 treatment.**

(a-b) Immunoblot analysis of LGR6 levels in cardiac tissue with and without Maresin1 treatment (n=5). (c-d) Immunoblot analysis of PGC-1α, NRF1 and NRF2 protein levels in cardiac tissue after Maresin1 treatment(n=5). Data are presented as mean ± SEM and analysed using a two-tailed unpaired Student's t-test. Statistical significance is indicated as follows: ns, no significant difference; **p* < 0.05; ***p* < 0.01; ****p* < 0.001.

**Supplementary Fig. 10:** **qPCR of mitochondrial biogenesis related genes after siLGR6 treatment.**

mRNA expression levels of PGC-1α, NRF1, and NRF2 in siNC and siLGR6 cardiomyocytes under Sham and HS conditions. Data are presented as mean ± SEM and analysed using two-way ANOVA and Tukey's multiple comparison tests. Statistical significance is indicated as follows: ns, no significant difference; **p* < 0.05; ***p* < 0.01; ****p* < 0.001.

**Supplementary Fig. 11:** **Maresin1 restores mitochondrial ETC complex protein expression under HS, while LGR6 knockdown abrogates this protective effect.**

Representative immunoblotting and quantitative analysis of mitochondrial electron transport chain (ETC) complex subunits in mice hearts exposed to HS. Protein levels of NDUFB8 (Complex I), SDHB (Complex II), UQCRC2 (Complex III), ATP5a1 (Complex V) and MTCO1 (Complex IV) were assessed by Western blotting. GAPDH served as the loading control. Data are presented as mean ± SEM and analysed using two-way ANOVA and Tukey's multiple comparison tests. Statistical significance is indicated as follows: ns, no significant difference; **p* < 0.05; ***p* < 0.01; ****p* < 0.001.

**Supplementary Fig. 12: The effect of maresin1/LGR6 axis on mitochondrial mtDNA damage in cardiomyocytes under HS**

Expression levels of (left) *Dloop*, (middle) *mt-Nd1*, and (right) *mt-Nd2* in H9c2 cardiomyocytes following Maresin1 treatment and LGR6 knockdown under HS. Data are presented as mean ± SEM and analyzed using one-way ANOVA with Tukey's post hoc test. Statistical significance is indicated as follows: ns, no significant difference; **p* < 0.05; ***p* < 0.01; ****p* < 0.001.

**Supplementary Fig. 13:** **LGR6-dependent transcriptional regulation of mitochondrial biogenesis-related genes under heatstroke conditions.**

(a) Schematic diagram of the experimental workflow. (B) Dual-luciferase reporter assays were performed to assess the promoter activities of PGC-1α, NRF1, and NRF2 in cells transfected with si-LGR6 and treated with Maresin1 (50 or 100 nM) under heatstroke conditions. Data are presented as mean ± SEM. Statistical significance is indicated as follows: ns, no significant difference; ****p* < 0.001.

**Supplementary Fig. 14: Assessment of mitochondrial biogenesis, stress response, apoptosis, and mitochondrial dynamics following LGR6 knockdown.**

Expression levels of mitochondrial biogenesis-related genes (*Tfam, Polg*), mitochondrial stress response markers (*Atf4, Ddit3/Chop*), apoptosis-related genes (*Bax, Bcl2*), and mitochondrial dynamics regulators (*Dnm1l/Drp1, Mfn1*) were analyzed following si-NC or si-LGR6 treatment. Statistical significance is indicated as follows: ns, no significant difference; **p* < 0.05; ***p* < 0.01; ****p* < 0.001.

**Supplementary Tables**

**Supplementary Table 1：Primers used for RT-qPCR analysis in this study.**

| Gene name | Primer | Sequences (5’-3’) |
| --- | --- | --- |
| Mouse LGR6 | Forward | CGTACTCTGTAACGGGCTGG |
|  | Reverse | GGGCTCCATACTCAGCAGAC |
| Mouse Nppa | Forward | AACCTGCAGACCACCTGGA |
|  | Reverse | ATCTATCGGAGGGGTCCCAG |
| Mouse Nppb | Forward | TTCGGTCTCAAGGCAC |
|  | Reverse | TTACAGCCCAAACGACTGAC |
| Mouse 𝛽-actin | Forward | CACAGAGCCTCGCCTTTGC |
|  | Reverse | AATCCTTCTGACCCATGCCC |
| Mouse PGC-1 𝛼 | Forward | AGTGGTGTAGCGACCAATCG |
|  | Reverse | GGGCAATCCGTCTTCATCCA |
| Mouse NRF1 | Forward | ACGGCCTCATGTGTTTGAGT |
|  | Reverse | ACTCGCGTCGTGTACTCATC |
| Mouse NRF2 | Forward | AGAGTGATGGTTGCCCACTT |
|  | Reverse | GCGTGCTCAGAAACCTCCTT |
| Rat Dloop | Forward | TGAAATCAACAACCCGCCCA |
|  | Reverse | TGATGGCCCTGAAGTAAGAACC |
| Rat mt-Nd1 | Forward | ATACGCCGCAGGACCATTC |
|  | Reverse | TGGTCATATCGAAAACGGGGG |
| Rat mt-Nd2 | Forward | CGCCTGAGGAGGACTTAACC |
|  | Reverse | TGCGAGTGAGAGTGTGTTGA |
| Rat 18SRNA | Forward | TCCGATAACGAACGAGAC |
|  | Reverse | CTAAGGGCATCACAGACC |
| Mouse Tfam | Forward | ATGCAGACGGAGCCATAGTG |
|  | Reverse | CTGGGAGAAAGGAGGGTTGC |
| Mouse Polg | Forward | GAGCTGTTCGTCAAAGGCAG |
|  | Reverse | GTGGGGACACCTCTCCAAGA |
| Mouse Atf4 | Forward | AGCCTGACTCTGCTGCTTAC |
|  | Reverse | TGGGCTCATACAGATGCCAC |
| Mouse Ddit3 | Forward | AACAGAGGTCACACGCACAT |
|  | Reverse | ACTTTCCGCTCGTTCTCCTG |
| Mouse Bax | Forward | GGAGATGAACTGGACAGCAATA |
|  | Reverse | GAAGTTGCCATCAGCAAACAT |
| Mouse Bcl2 | Forward | GCTGGGGATGACTTCTCTCG |
|  | Reverse | CCACAATCCTCCCCCAGTTC |
| Mouse Ddm1l | Forward | CCGGCTTGACAGTGAGTACA |
|  | Reverse | AGGCTACAGTGGTGGTCATC |
| Mouse Mfn1 | Forward | CCCTCTTTCGGGAGGATGAGAC |
|  | Reverse | CAGCGGACACTCACTCCGT |

**Supplementary Table 2: Information of primary antibodies.**

| Antibody | Company | Cat no. | Source | Application |
| --- | --- | --- | --- | --- |
| PGC-1 𝛼 | Proteintech | 66369-1-lg | Mouse | IF |
| PGC-1𝛼 | Affinity | AF5395 | Rabbit | WB |
| NRF1 | Proteintech | 12482-1-AP | Rabbit | IF |
| NRF2 | Proteintech | 80593-1-RR | Rabbit | IF |
| NRF1 | Affinity | #AF5298 | Rabbit | IF |
| LGR6 | Abcam | Ab126747 | Rabbit | WB, IF |
| 𝛽-actin | Proteintech | HRP-66009 | Mouse | WB |
| Tnnt2 | Invitrogen | MA5-12960 | Mouse | IF |
| AF-647-LGR6 | Signalway | C56659 | Mouse | Flow Cytometry |
| AF-488-Tnnt2 | Abcam | Ab314676 | Rabbit | Flow Cytometry |
| BV421-CD31 | Biolegend | 102423 | Mouse | Flow Cytometry |
| PE-CD45 | ThermoFisher | 12-0452-82 | Mouse | Flow Cytometry |
| PE/cy7-CD90 | Biolegend | 202518 | Mouse | Flow Cytometry |
| ATP5A1 | HUABIO | ET1703-53 | Rabbit | WB |
| UQCRC2 | HUABIO | HA721872 | Rabbit | WB |
| MTCO1 | HUABIO | HA500517 | Rabbit | WB |
| SDHB | HUABIO | ET1706-30 | Rabbit | WB |
| NDUFB8 | HUABIO | ET7108-25 | Rabbit | WB |
| GAPDH | Proteintech | 10494-1-AP | Rabbit | WB |
